# Delayed skeletal development and IGF-1 deficiency in a mouse model of lysinuric protein intolerance

**DOI:** 10.1242/dmm.050118

**Published:** 2023-08-17

**Authors:** Bridget M. Stroup, Xiaohui Li, Sara Ho, Haonan Zhouyao, Yuqing Chen, Safa Ani, Brian Dawson, Zixue Jin, Ronit Marom, Ming-Ming Jiang, Isabel Lorenzo, Daniel Rosen, Denise Lanza, Nathalie Aceves, Sara Koh, John R. Seavitt, Jason D. Heaney, Brendan Lee, Lindsay C. Burrage

**Affiliations:** ^1^ Department of Molecular and Human Genetics, Baylor College of Medicine, Houston, TX 77030, USA; ^2^ Texas Children's Hospital, Houston, TX 77030, USA; ^3^ Department of Pathology and Immunology, Baylor College of Medicine, Houston, TX 77030, USA; ^4^ Rice University, Houston, TX 77005, USA

**Keywords:** Osteoporosis, Osteoblast, IGF-1, Lysinuric protein intolerance, *Slc7a7*, Arginine

## Abstract

*SLC7A7* deficiency, or lysinuric protein intolerance (LPI), causes loss of function of the y^+^LAT1 transporter critical for efflux of arginine, lysine and ornithine in certain cells. LPI is characterized by urea cycle dysfunction, renal disease, immune dysregulation, growth failure, delayed bone age and osteoporosis. We previously reported that *Slc7a7* knockout mice (C57BL/6×129/SvEv F2) recapitulate LPI phenotypes, including growth failure. Our main objective in this study was to characterize the skeletal phenotype in these mice. Compared to wild-type littermates, juvenile *Slc7a7* knockout mice demonstrated 70% lower body weights, 87% lower plasma IGF-1 concentrations and delayed skeletal development. Because poor survival prevents evaluation of mature knockout mice, we generated a conditional *Slc7a7* deletion in mature osteoblasts or mesenchymal cells of the osteo-chondroprogenitor lineage, but no differences in bone architecture were observed. Overall, global *Slc7a7* deficiency caused growth failure with low plasma IGF-1 concentrations and delayed skeletal development, but *Slc7a7* deficiency in the osteoblastic lineage was not a major contributor to these phenotypes. Future studies utilizing additional tissue-specific *Slc7a7* knockout models may help dissect cell-autonomous and non-cell-autonomous mechanisms underlying phenotypes in LPI.

## Teaser

**Summary:** Global *Slc7a7* deficiency causes postnatal growth failure with low plasma IGF-1 concentrations and delayed skeletal development, but *Slc7a7* deficiency in the osteoblastic lineage is not a major contributor to these phenotypes.

## INTRODUCTION

Lysinuric protein intolerance [LPI; Online Mendelian Inheritance in Man (OMIM) #222700] is an inborn error of cationic amino acid transport caused by biallelic pathogenic variants in *SLC7A7* that results in loss of function of the y^+^LAT1 transporter (Borsani et al., 1999; Torrents et al., 1999). The heteromeric y^+^LAT1 transporter, composed of a light subunit (encoded by *SLC7A7*) and a heavy subunit (encoded by *SLC3A2*), catalyzes the transmembrane efflux of arginine, lysine and ornithine in certain epithelial and non-epithelial cell types (Broer, 2008; Broer and Gauthier-Coles, 2022; Palacin et al., 2004). LPI is a severe multi-system disorder characterized by growth failure (Awrich et al., 1975; Goto et al., 1984; Carpenter et al., 1985; Nagata et al., 1987; Takada et al., 1987; Parini et al., 1991; Svedstrom et al., 1993; Parenti et al., 1995; Parsons et al., 1996; Kamoda et al., 1998; Korman et al., 2002; Moosa et al., 2005; Esposito et al., 2006; Gomez et al., 2006; Ogier de Baulny et al., 2012; Ko et al., 2012; Guzel-Ozanturk et al., 2013; Posey et al., 2014; Evelina et al., 2015; Bijarnia-Mahay et al., 2016; Deogaonkar and Shah, 2016; Noguchi et al., 2016; Mauhin et al., 2017; Stanley et al., 2017; Zhang and Cao, 2017; Olgac et al., 2020; Kang et al., 2019; Aljishi et al., 2020; Contreras et al., 2021; Andrews et al., 2021; Al-Qattan et al., 2021; Hashmi and Ahmed, 2022; Lee et al., 2022; Alqarajeh et al., 2020; Nicolas et al., 2016), short stature (Awrich et al., 1975; Goto et al., 1984; Carpenter et al., 1985; Nagata et al., 1987; Takada et al., 1987; Parini et al., 1991; Parenti et al., 1995; Parsons et al., 1996; Kamoda et al., 1998; Korman et al., 2002; Moosa et al., 2005; Esposito et al., 2006; Gomez et al., 2006; Ogier de Baulny et al., 2012; Ko et al., 2012; Guzel-Ozanturk et al., 2013; Posey et al., 2014; Evelina et al., 2015; Bijarnia-Mahay et al., 2016; Deogaonkar and Shah, 2016; Noguchi et al., 2016; Mauhin et al., 2017; Stanley et al., 2017; Zhang and Cao, 2017; Olgac et al., 2020; Kang et al., 2019; Aljishi et al., 2020; Contreras et al., 2021; Andrews et al., 2021; Al-Qattan et al., 2021; Hashmi and Ahmed, 2022; Lee et al., 2022; Alqarajeh et al., 2020; Nicolas et al., 2016; Rajantie et al., 1980a; Tanner et al., 2007a; Simell et al., 1975, 1986; No author listed, 1986; Aoki et al., 2001; Niinikoski et al., 2011), secondary urea cycle dysfunction (Awrich et al., 1975; Goto et al., 1984; Carpenter et al., 1985; Nagata et al., 1987; Takada et al., 1987; Parini et al., 1991; Svedstrom et al., 1993; Parenti et al., 1995; Parsons et al., 1996; Kamoda et al., 1998; Korman et al., 2002; Moosa et al., 2005; Esposito et al., 2006; Ogier de Baulny et al., 2012; Ko et al., 2012; Guzel-Ozanturk et al., 2013; Posey et al., 2014; Bijarnia-Mahay et al., 2016; Deogaonkar and Shah, 2016; Noguchi et al., 2016; Mauhin et al., 2017; Zhang and Cao, 2017; Olgac et al., 2020; Kang et al., 2019; Aljishi et al., 2020; Al-Qattan et al., 2021; Lee et al., 2022; Nicolas et al., 2016; Rajantie et al., 1980a; Tanner et al., 2007a,b, 2008; No author listed, 1986; Aoki et al., 2001; Niinikoski et al., 2011; Mykkanen et al., 2000; Siri et al., 2022; Kakisaka et al., 2022; Maines et al., 2013; Benninga et al., 2007; Kerem et al., 1993; Parto et al., 1993a; Lukkarinen et al., 1999; Simell et al., 1986), delayed bone age and osteoporosis (Awrich et al., 1975; Goto et al., 1984; Carpenter et al., 1985; Svedstrom et al., 1993; Parenti et al., 1995; Parsons et al., 1996; Korman et al., 2002; Esposito et al., 2006; Gomez et al., 2006; Guzel-Ozanturk et al., 2013; Posey et al., 2014; Evelina et al., 2015; Zhang and Cao, 2017; Olgac et al., 2020; Aljishi et al., 2020; Andrews et al., 2021; Al-Qattan et al., 2021; Lee et al., 2022; Alqarajeh et al., 2020; Rajantie et al., 1980b; Simell et al., 1975, 1986; No author listed, 1986; Aoki et al., 2001; Niinikoski et al., 2011; Kerem et al., 1993; Parto et al., 1993a; Tanner et al., 2007b; Avci Durmusalioglu et al., 2021), in addition to neurological complications (Torrents et al., 1999; Awrich et al., 1975; Takada et al., 1987; Parini et al., 1991; Svedstrom et al., 1993; Parenti et al., 1995; Kamoda et al., 1998; Moosa et al., 2005; Guzel-Ozanturk et al., 2013; Bijarnia-Mahay et al., 2016; Deogaonkar and Shah, 2016; Noguchi et al., 2016; Mauhin et al., 2017; Olgac et al., 2020; Kang et al., 2019; Aljishi et al., 2020; Al-Qattan et al., 2021; Hashmi and Ahmed, 2022; Lee et al., 2022; Alqarajeh et al., 2020; Aoki et al., 2001; Benninga et al., 2007; Kerem et al., 1993; Noguchi et al., 2000; Esteve et al., 2017), renal disease (Parenti et al., 1995; Parsons et al., 1996; Esposito et al., 2006; Guzel-Ozanturk et al., 2013; Deogaonkar and Shah, 2016; Noguchi et al., 2016; Mauhin et al., 2017; Lee et al., 2022; Nicolas et al., 2016; Benninga et al., 2007; Tanner et al., 2007b, 2008; Lukkarinen et al., 1999; Esteve et al., 2017; Pitkanen et al., 2018; Miller et al., 2022), pulmonary disease (Svedstrom et al., 1993; Parenti et al., 1995; Parsons et al., 1996; Kamoda et al., 1998; Guzel-Ozanturk et al., 2013; Deogaonkar and Shah, 2016; Mauhin et al., 2017; Stanley et al., 2017; Zhang and Cao, 2017; Aljishi et al., 2020; Nicolas et al., 2016; Aoki et al., 2001; Kerem et al., 1993; Lukkarinen et al., 1999; Tanner et al., 2017; Valimahamed-Mitha et al., 2015; Ceruti et al., 2007; Santamaria et al., 2004), immunodeficiency (Nagata et al., 1987; Parsons et al., 1996; Kamoda et al., 1998; Ko et al., 2012; Stanley et al., 2017; Aoki et al., 2001; Tanner et al., 2007b; Lukkarinen et al., 1999; Yoshida et al., 1995) and inflammatory disorders, such as hemophagocytic lymphohistiocytosis (Carpenter et al., 1985; Svedstrom et al., 1993; Parenti et al., 1995; Korman et al., 2002; Ko et al., 2012; Guzel-Ozanturk et al., 2013; Evelina et al., 2015; Bijarnia-Mahay et al., 2016; Deogaonkar and Shah, 2016; Noguchi et al., 2016; Mauhin et al., 2017; Stanley et al., 2017; Zhang and Cao, 2017; Olgac et al., 2020; Kang et al., 2019; Aljishi et al., 2020; Contreras et al., 2021; Al-Qattan et al., 2021; Rajantie et al., 1980b; Maines et al., 2013; Benninga et al., 2007; Kerem et al., 1993; Parto et al., 1993a,b; Lukkarinen et al., 1999; Valimahamed-Mitha et al., 2015; Matsukawa et al., 2022) and early-onset autoimmune diseases (Svedstrom et al., 1993; Parsons et al., 1996; Kamoda et al., 1998; Noguchi et al., 2016; Aljishi et al., 2020; Contreras et al., 2021; Lee et al., 2022; Aoki et al., 2001; Maines et al., 2013; Parto et al., 1993a, 1994; Lukkarinen et al., 1999; Liu et al., 2022).

Diminished intestinal absorption and renal reabsorption of the cationic amino acids lead to reduced availability of the urea cycle intermediates, arginine and ornithine, and contribute to the secondary urea cycle dysfunction in LPI (Ogier de Baulny et al., 2012; Rajantie, 1981; Rajantie et al., 1980a, 1983a). Moreover, treatment regimens that aim to reduce the risk for hyperammonemia using dietary protein restriction, L-citrulline supplementation and nitrogen-scavenging agents target this secondary urea cycle dysfunction (Ogier de Baulny et al., 2012; Simell et al., 1986; Rajantie et al., 1983b). Cationic amino acid deficiency may also impair the secretion of growth hormone (GH) and insulin-like growth factor-1 (IGF-1) and is hypothesized to be an underlying mechanism of growth failure in LPI (Ogier de Baulny et al., 2012). However, studies investigating the therapeutic efficacy of supplementation with L-lysine or L-citrulline (Awrich et al., 1975; Al-Qattan et al., 2021; Rajantie et al., 1980b; Tanner et al., 2007a) and recombinant growth hormone therapy (Awrich et al., 1975; Goto et al., 1984; Esposito et al., 2006; Evelina et al., 2015; Al-Qattan et al., 2021; Tanner et al., 2007a) for growth failure in individuals with LPI yield inconsistent outcomes. Regardless, treatments that target the underlying mechanisms of other complications in LPI have not been optimized owing to our limited understanding of the pathophysiology of this disorder. Thus, generation of viable mouse models of *Slc7a7* deficiency is essential to advance our understanding of the disease mechanisms in order to develop more targeted therapies for individuals with LPI.

We recently described the generation of our global *Slc7a7* knockout mouse model (*Slc7a7^em1Lbu/em1Lbu^*, hereafter referred to as *Slc7a7^Lbu/Lbu^*) that recapitulates aspects of the human disorder, including growth failure, delayed development in multiple tissues (i.e. L_4_ vertebrae, lungs and kidney) and proximal tubular dysfunction (Stroup et al., 2020). Compared to the first reported global knockout mouse model [C57BL/6 background, gene trap allele *Slc7a7^Gt(OST41878)Lex^*], which generated only two surviving homozygous knockout mice for postnatal studies owing to early lethality (Sperandeo et al., 2007), we reported severe postnatal growth failure in *Slc7a7^Lbu/Lbu^* mice (C57BL/6×129/SvEv F2 background) compared to wild-type (WT) littermates in a large cohort of mice (Stroup et al., 2020). Consistent with the hypothesis that growth failure in LPI may be driven by cationic amino acid deficiency and subsequent perturbations in the GH/IGF-1 axis, the *Slc7a7^Lbu/Lbu^* knockout mouse model exhibits the classical biochemical phenotype (low plasma concentrations and increased urinary excretion of the cationic amino acids) and reduced hepatic expression of *Igf1* during the postnatal period (Stroup et al., 2020). Owing to the delayed skeletal development in *Slc7a7^Lbu/Lbu^* mice, we were unable to quantify trabecular bone mass using micro-computed tomography (micro-CT) to test whether this mouse model has the osteoporosis phenotype associated with LPI (Stroup et al., 2020). Single-cell RNA-sequencing studies have demonstrated that *Slc7a7* is expressed in osteoblasts (Yoshioka et al., 2021), and, interestingly, reduced *Slc7a7* expression in ST2 cells (stable cell line of murine stromal cells) hindered WNT-induced osteoblast differentiation by preventing glutamine uptake in response to WNT-induced β-catenin signaling (Shen et al., 2021, 2022). Although this *in vitro* study illustrates the involvement of *Slc7a7* in ST2-cell-derived osteoblast differentiation (Shen et al., 2021), *in vivo* studies using conditional *Slc7a7* knockout mouse models are needed to assess the relative contribution of *Slc7a7* expression in osteoblasts and other tissues to growth failure and skeletal development.

In this report, we expand our previous work to further characterize the growth failure and skeletal phenotypes in mouse models of LPI. First, we demonstrate that *Slc7a7^Lbu/Lbu^* mice exhibited severe postnatal growth failure and reduced plasma IGF-1 concentrations compared to those of WT embryos, whereas *Slc7a7^Lbu/Lbu^* embryos exhibited mild intrauterine growth restriction (IUGR) and similar plasma IGF-1 concentrations to those of WT embryos. Postnatal growth failure, delayed skeletal development and reduced survival prevent studies of bone mineralization and osteoporosis in skeletally mature mice with *Slc7a7* deficiency; therefore, we generated a conditional *Slc7a7* mouse model. Given the growth failure and delayed skeletal development in *Slc7a7^Lbu/Lbu^* mice and the role of *Slc7a7* in osteoblast differentiation in cell studies (Shen et al., 2021, 2022), we hypothesized that loss of *Slc7a7* in osteoblasts might impair growth and skeletal development and contribute to osteoporosis in LPI. However, the bone architecture and growth remained undisrupted with specific *Slc7a7* deletion in mature osteoblasts and in mesenchymal cells of the osteo-chondroprogenitor lineage utilizing an Ocn-driven Cre transgene and a Prx1-driven Cre transgene, respectively. Thus, our data suggest that *Slc7a7* deficiency in osteoblasts does not contribute to the growth failure, delayed skeletal development or osteoporosis phenotypes in LPI.

## RESULTS

### IUGR and postnatal growth failure in *Slc7a7^Lbu/Lbu^* mice

**Fig. 1.**
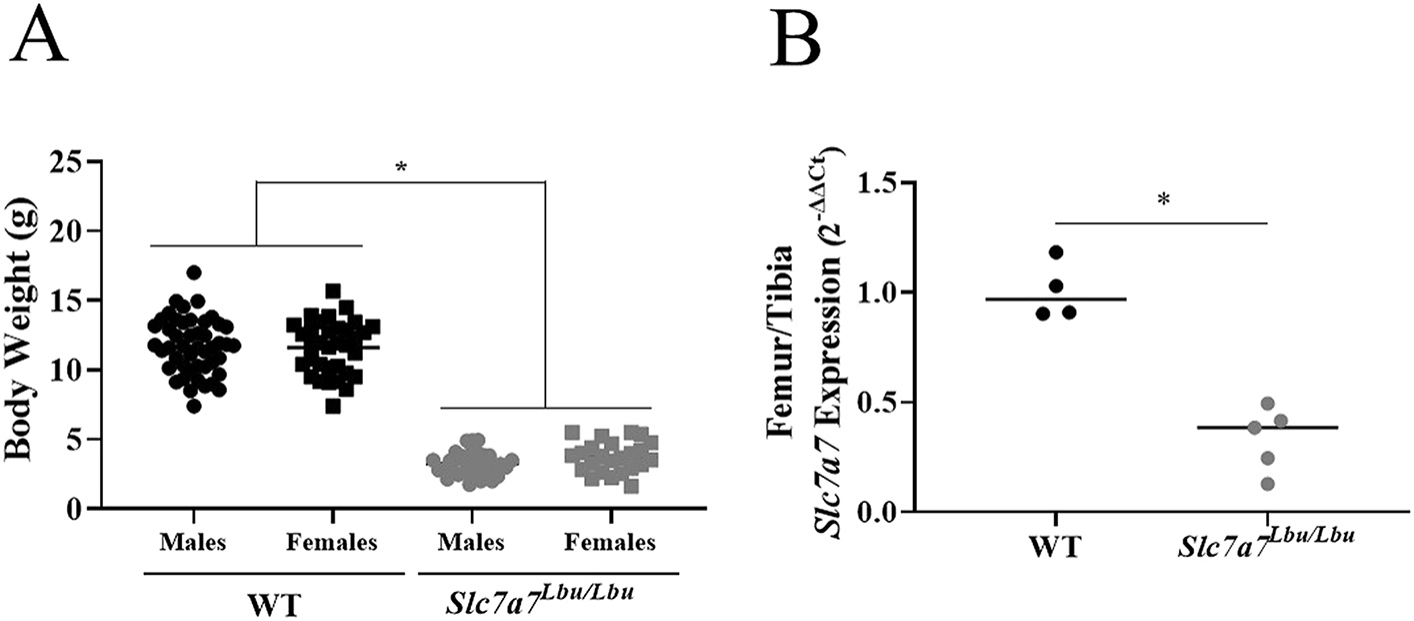
**Growth failure and reduced *Slc7a7* expression in combined femurs and tibias of *Slc7a7^Lbu/Lbu^* versus WT mice.** Male and female WT and *Slc7a7^Lbu/Lbu^* littermates were weighed prior to tissue harvests at 14-18 days of age. The lower extremities (femur/tibias were combined and bone marrow was flushed) from WT and *Slc7a7^Lbu/Lbu^* mice were harvested at postnatal day (P)14-P18. *Slc7a7* expression was evaluated using quantitative PCR. (A) Regardless of sex, *Slc7a7^Lbu/Lbu^* mice demonstrated significantly lower body weights than those of WT mice (two-way ANOVA; genotype, *P<0.0001; sex, *P*=0.30; genotype by sex, *P*=0.28). Sample sizes included the following: WT (males, *n*=47; females, *n*=34) and *Slc7a7^Lbu/Lbu^* (males, *n*=35; females, *n*=24). (B) *Slc7a7^Lbu/Lbu^* mice (*n*=5) demonstrated significantly lower expression of *Slc7a7* in the femurs/tibias compared to WT mice (*n*=4, unpaired two-tailed *t*-test, *P=0.0002). WT, wild type.

Consistent with the findings of growth failure in *Slc7a7^Lbu/Lbu^* mice in our previous study (*n*=95), we observed severe growth failure in male and female *Slc7a7^Lbu/Lbu^* mice versus WT littermates in the expanded dataset comprised of 140 mice (Fig. 1A) (Stroup et al., 2020). To complement the skeletal focus of the present study, we sought to confirm reduced expression of *Slc7a7* in the combined femurs and tibias of *Slc7a7^Lbu/Lbu^* versus WT mice. Similar to our previous findings of reduced *Slc7a7* expression in multiple tissues (i.e. small intestines, kidney, spleen, liver, lungs and calvaria) (Stroup et al., 2020), we observed reduced expression of *Slc7a7* in the combined femurs and tibias of *Slc7a7^Lbu/Lbu^* versus WT mice (Fig. 1B).

**Fig. 2.**
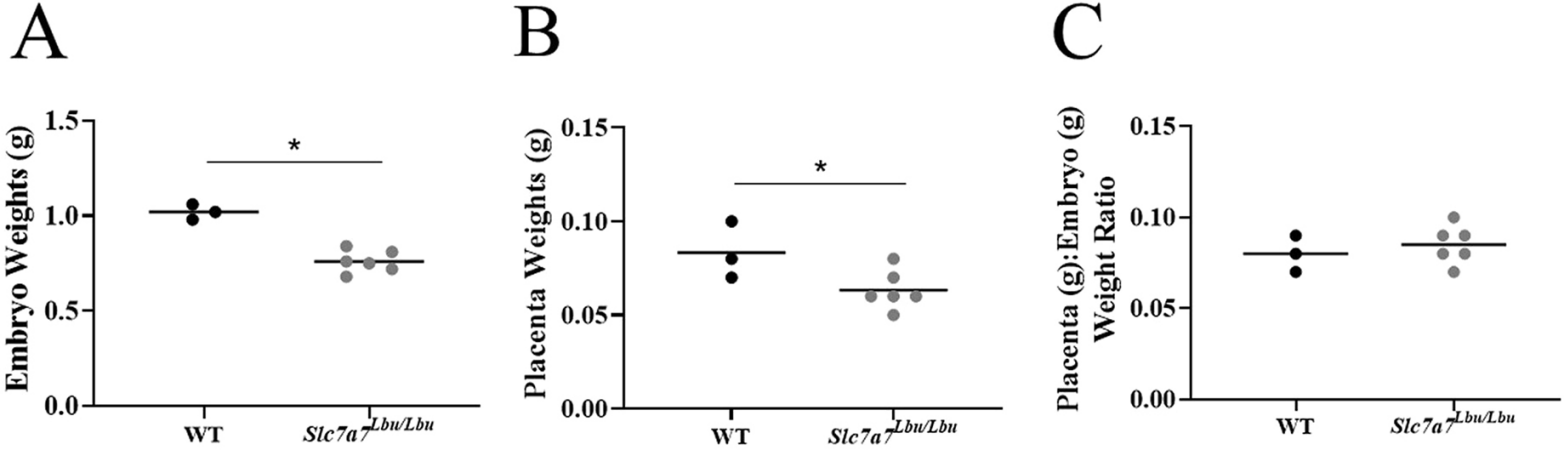
**IUGR in *Slc7a7^Lbu/Lbu^* embryos.** Timed matings were performed, and embryos and placentas were harvested and weighed at embryonic day (E)17.5. (A) *Slc7a7^Lbu/Lbu^* weighed significantly less than WT embryos (*P=0.0002). (B) Placentas from *Slc7a7^Lbu/Lbu^* embryos weighed significantly less than placentas from WT embryos (*P=0.049). (C) Placenta (g) to body weight (g) ratios were similar between WT and *Slc7a7^Lbu/Lbu^* embryos (*P*=0.52). IUGR, intrauterine growth restriction; WT, wild type. Unpaired two-tailed *t*-tests were used for statistical comparisons with *n*=3-6 per group.

We previously demonstrated IUGR in *Slc7a7*-deficient mouse embryos (*Slc7a7^Bay/Bay^*, C57BL/6NJ background) (Stroup et al., 2020). To determine whether placenta size or pathology might contribute to this IUGR, we evaluated the placenta weight and pathology in *Slc7a7^Lbu/Lbu^* and WT embryos (C57BL/6×129/SvEv F2 background). Consistent with previous reports (Stroup et al., 2020; Sperandeo et al., 2007), we observed significant reductions in the body weights of *Slc7a7^Lbu/Lbu^* embryos compared to those of WT embryos at embryonic day (E)17.5, indicating IUGR with global *Slc7a7* deficiency (Fig. 2A). Although the placentas from *Slc7a7^Lbu/Lbu^* embryos weighed significantly less than those from WT embryos (Fig. 2B), the placenta-to-embryo weight ratios were similar (Fig. 2C). Furthermore, no gross abnormalities in the architecture of Hematoxylin and Eosin (H&E)-stained placenta sections were noted among WT and *Slc7a7^Lbu/Lbu^* embryos (Fig. S1). Interestingly, when compared to age-matched WT controls, *Slc7a7^Lbu/Lbu^* mice demonstrated more severe growth failure at postnatal day (P)14-P18 than *Slc7a7^Lbu/Lbu^* embryos at E17.5 (70% versus 25% lower body weights, respectively). Taken together, these findings suggest a prenatal onset of growth failure in *Slc7a7^Lbu/Lbu^* mice that worsens in the postnatal period.

### Reduced plasma IGF-1 concentrations in *Slc7a7^Lbu/Lbu^* mice

**Fig. 3.**
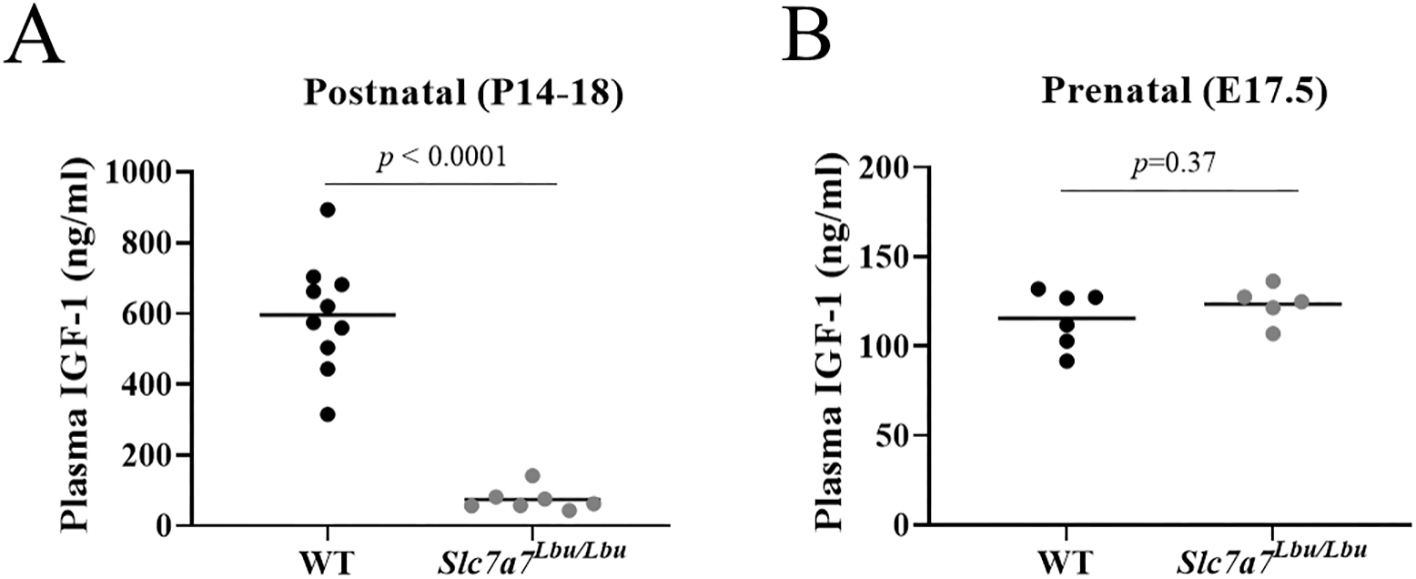
**Plasma IGF-1 concentrations in WT versus *Slc7a7^Lbu/Lbu^* mice during the prenatal and postnatal periods.** Plasma was obtained from WT and *Slc7a7^Lbu/Lbu^* mice at E17.5 and P14-P18. Using an enzyme-linked immunoassay, plasma IGF-1 concentrations were evaluated. (A) *Slc7a7^Lbu/Lbu^* mice demonstrated significantly lower plasma IGF-1 concentrations than those of WT mice at P14-P18 (*n*=7-10/genotype). (B) Similar plasma IGF-1 concentrations were observed in WT and *Slc7a7^Lbu/Lbu^* mice at E17.5 (*n*=5-6/genotype). IGF-1, insulin-like growth factor 1; WT, wild type. Unpaired two-tailed *t*-tests were used for statistical comparisons.

Given the reports of reduced circulating concentrations of GH and IGF-1 in children with LPI that also suffer from growth failure (Goto et al., 1984; Esposito et al., 2006; Evelina et al., 2015; Al-Qattan et al., 2021; Niinikoski et al., 2011), we assessed the plasma concentrations of IGF-1 in our *Slc7a7^Lbu/Lbu^* mouse model. Our hypothesis was twofold. First, we hypothesized that *Slc7a7^Lbu/Lbu^* mice would demonstrate reduced plasma IGF-1 concentrations compared to WT mice postnatally, considering that *Slc7a7^Lbu/Lbu^* mice exhibit arginine deficiency (Stroup et al., 2020) and arginine is required for the hepatic secretion of IGF-1 (Tsugawa et al., 2019; Oh et al., 2017). Second, we hypothesized that *Slc7a7^Lbu/Lbu^* embryos would demonstrate similar plasma IGF-1 concentrations to WT embryos as we predicted that *Slc7a7^Lbu/Lbu^* embryos likely have normal plasma concentrations of arginine. Consistent with our hypothesis, we observed reduced plasma concentrations of IGF-1 in *Slc7a7^Lbu/Lbu^* mice (aged P14-P18), but not in *Slc7a7^Lbu/Lbu^* embryos (aged E17.5) compared to age-matched WT controls (Fig. 3). Because *Slc7a7^Lbu/Lbu^* mice showed greater postnatal growth failure with low plasma IGF-1 concentrations compared to the moderate IUGR in *Slc7a7^Lbu/Lbu^* embryos with normal plasma IGF-1 concentrations, subsequent skeletal phenotyping studies were only performed in P14-P18 WT and *Slc7a7^Lbu/Lbu^* mice.

### Delayed skeletal development at multiple sites in male and female *Slc7a7^Lbu/Lbu^* mice

**Fig. 4.**
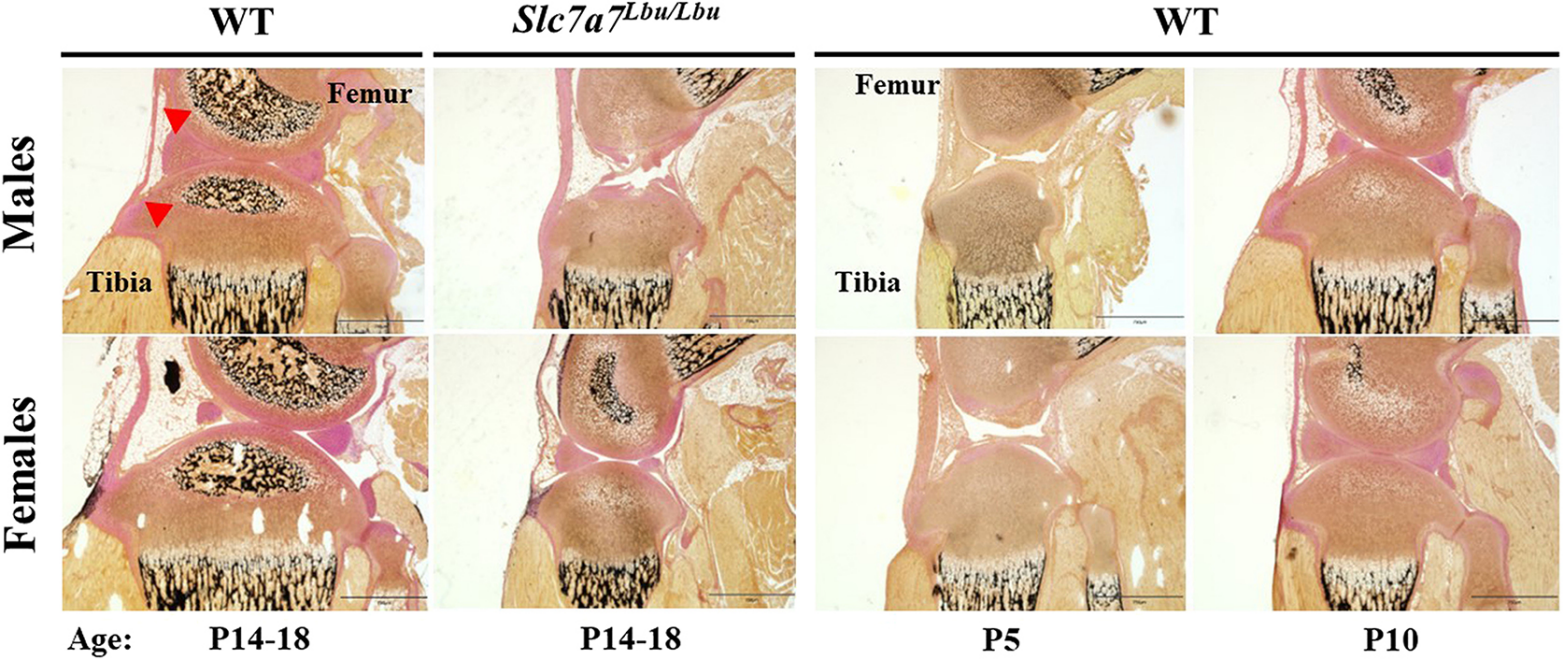
**Delayed skeletal development in the tibias and femurs of *Slc7a7^Lbu/Lbu^* mice compared to those of WT mice.** Representative images of von Kossa-stained lower extremities of male and female *Slc7a7^Lbu/Lbu^* and WT mice (aged P14-P18) (left two columns). Representative images of von Kossa-stained lower extremities of male and female WT mice at ages P5 and P10 (right two columns) are shown to demonstrate the noticeable delay in skeletal development in the *Slc7a7^Lbu/Lbu^* mice. Samples sizes included 2-3 per genotype per sex. Similar to male and female WT mice at P5 and P10, male and female *Slc7a7^Lbu/Lbu^* mice (aged P14-P18) demonstrated absent or smaller secondary ossification centers in the proximal tibias and distal femurs compared to those of sex- and age-matched WT littermates. Scale bars: 750 µm. Red arrowheads indicate the secondary ossification centers. WT, wild type.

**Fig. 5.**
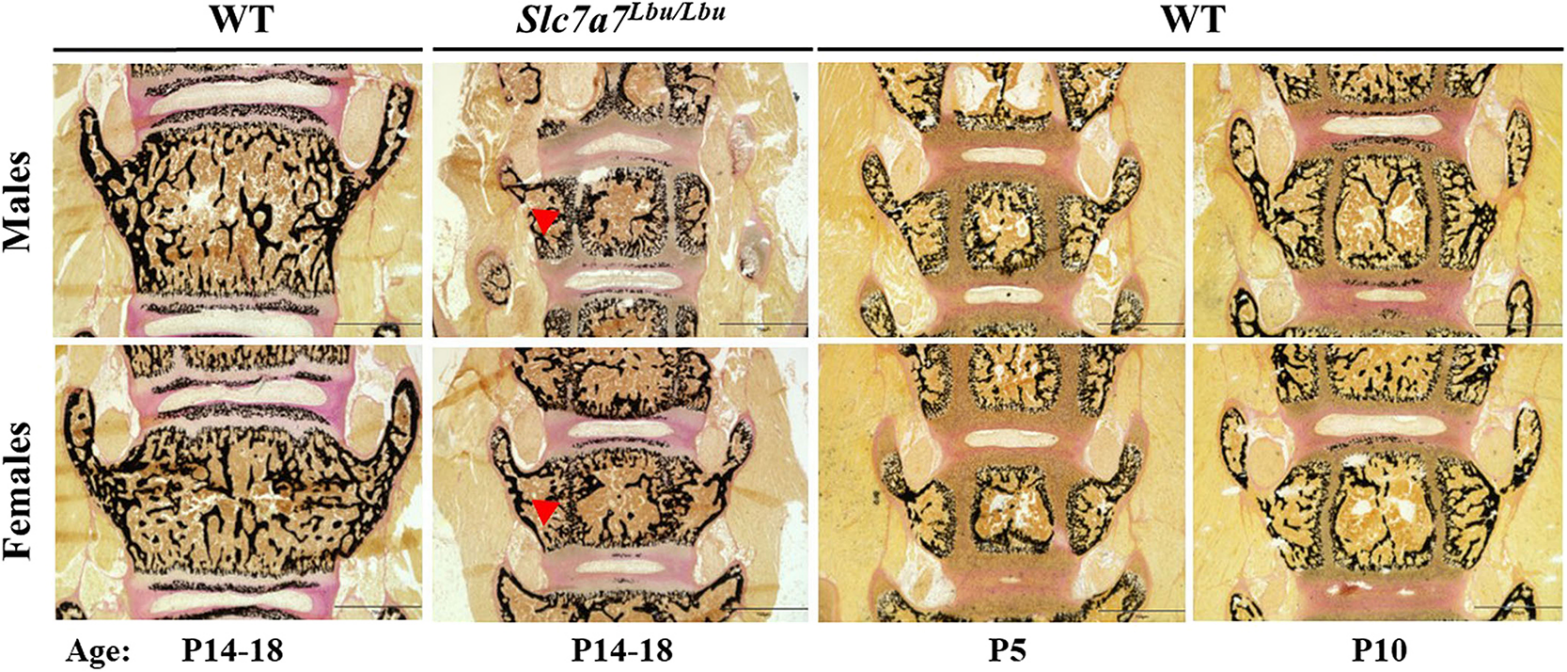
**Delayed skeletal development in the L_4_ vertebrae of *Slc7a7^Lbu/Lbu^* versus WT mice.** Representative images of von Kossa-stained L_4_ vertebrae sections of male and female WT mice (aged P5, P10 or P14-P18) and *Slc7a7^Lbu/Lbu^* mice (aged P14-P18) were visualized. Delayed development in the L_4_ vertebrae of P14-18 *Slc7a7^Lbu/Lbu^* mice was observed, as evidenced by the retention of the growth plates within the vertebral body. Samples sizes included 2-3 per genotype. Scale bars: 750 μm. Red arrowheads indicate the retained growth plates within the L_4_ vertebral bodies in the *Slc7a7^Lbu/Lbu^* mice. WT, wild type.

To assess skeletal architecture and development, we performed von Kossa staining in lower-extremity and L_4_ vertebrae sections of male and female WT mice (aged P5, P10 or P14-P18) and *Slc7a7^Lbu/Lbu^* mice (aged P14-P18). We observed delayed development in the femurs and tibias of male and female *Slc7a7^Lbu/Lbu^* mice compared to those of age-matched male and female WT mice, as evidenced by the femoral and tibial ossification centers that were either absent or smaller in size and, therefore, appeared more similar to those of P5-P10 WT mice than those of P14-P18 age-matched WT littermates (Fig. 4; Fig. S2). In addition, we observed delayed development in the L_4_ vertebrae of male and female *Slc7a7^Lbu/Lbu^* mice, compared to those of age-matched WT littermates, with the retention of the growth plates within the vertebral bodies (Fig. 5; Fig. S3).

Consistent with reports of reduced calcification rates in bone biopsies from two individuals with LPI and osteopenia or osteoporosis (Carpenter et al., 1985; No author listed, 1986), we previously reported that the *Slc7a7^Lbu/Lbu^* mice (aged P14-P18) might also exhibit a mineralization defect (Stroup et al., 2020). Although the skeletal radiographs of the lower extremities and spines of male and female *Slc7a7^Lbu/Lbu^* mice might appear less mineralized than those of age-matched WT littermates (Fig. S6), our findings of delayed skeletal development at multiple sites in *Slc7a7^Lbu/Lbu^* mice (Figs S4 and S5) hinder firm conclusions regarding the presence of a skeletal mineralization defect or the presence of abnormal osteoblast or osteoclast activity in this model using micro-CT (Stroup et al., 2020; Zenzes et al., 2019), radiography and histology/histomorphometry (Figs S4 and S5).

### Deletion of *Slc7a7* in osteoblasts does not promote skeletal defects

Given that recent *in vitro* studies have implicated *Slc7a7* in osteoblast differentiation (Shen et al., 2021, 2022), we hypothesized that *Slc7a7* deficiency in osteoblasts might cause osteoporosis. To test whether loss of *Slc7a7* impairs osteoblast differentiation or mineralization *in vitro*, we isolated calvaria osteoblasts from *Slc7a7^Lbu/Lbu^* and WT or heterozygous embryos at E18.5. Staining for alkaline phosphatase, a marker for osteoblast activity, after 7 days in culture was similar in both control and mutant genotypes (Fig. S7). Likewise, staining with Alizarin Red, a marker for mineralization, after 15 days in culture was similar in cells of control and mutant genotypes (Fig. S7). These results suggest that loss of *Slc7a7* does not affect osteoblast differentiation or mineralization *in vitro*.

Next, we assessed the impact of loss of *Slc7a7* in osteoblasts *in vivo*. The poor survival and growth failure in the *Slc7a7^Lbu/Lbu^* mouse model (Stroup et al., 2020) prevented evaluations of osteoporosis and bone mineralization in skeletally mature mice. Thus, we generated a conditional *Slc7a7* knockout mouse model [C57BL/6N-*Slc7a7^tm1c(EUCOMM)Wtsi^*; *Slc7a7^f/f^*] that harbors a conditional allele with loxP sites flanking exons 3 and 4 (ENSMUSE00000124135 and ENSMUSE00000124133 of Ensembl transcript ENSMUST00000000984; Fig. S8). Similar to our global knockout mouse model (*Slc7a7^Lbu/Lbu^*), deletion of exons 3 and 4 is predicted to result in a frameshift, premature stop codon and nonsense-mediated mRNA decay (Stroup et al., 2020). We selectively deleted *Slc7a7* in osteoblasts using the osteocalcin promoter-driven Cre transgene Tg(BGLAP-cre)1Clem (Ocn-Cre) to test whether *Slc7a7* deficiency in osteoblasts impairs bone formation *in vivo* in skeletally mature mice (Zhang et al., 2002).

To confirm the activity of the Cre transgene, we crossed mice harboring the Ocn-Cre transgene with mice harboring the *Rosa26* knock-in Cre reporter [*Gt(ROSA)26Sor^tm6(CAG-ZsGreen1)Hze^*; Ai6] (Madisen et al., 2010). As expected, we observed Ai6-expressing cells within the femur and tibia of Ocn-Cre^+^*;* Ai6^+^ mice, but not in those of Ai6^+^ mice (Fig. S9). Furthermore, neither Ai6^+^ nor Ocn-Cre*^+^;* Ai6^+^ mice had Ai6-expressing cells in the kidney (Fig. S9) or liver (Fig. S10). Consistent with this finding, relative DNA copy number of exon 3 of *Slc7a7* was significantly lower in Ocn-Cre^+^*; Slc7a7^f/f^* versus *Slc7a7^f/f^* primary calvaria-derived osteoblasts (Fig. S11A). In addition, gene expression of *Slc7a7* in the femurs was reduced in Ocn-Cre^+^*; Slc7a7^f/f^* versus *Slc7a7^f/f^* mice (Fig. S11B).

At 6 months of age, Ocn-Cre^+^*; Slc7a7^f/f^* mice demonstrated similar growth to that of sex-matched *Slc7a7^f/f^* littermates (Fig. S12). To assess bone microarchitecture in this osteoblast-specific *Slc7a7* knockout mouse model, we performed micro-CT in the femurs and L_4_ vertebrae of male and female Ocn-Cre^+^*; Slc7a7^f/f^* and *Slc7a7^f/f^* mice. The femurs of Ocn-Cre^+^*; Slc7a7^f/f^* mice exhibited similar metrics related to size (i.e. length, apical diameter and mediolateral diameter), in addition to cortical and trabecular architecture, to those of sex-matched *Slc7a7^f/f^* littermates (Table 1). Likewise, trabecular microarchitecture outcomes were similar in the L_4_ vertebrae of Ocn-Cre^+^*; Slc7a7^f/f^* and *Slc7a7^f/f^* mice, regardless of sex (Table 1). Consistent with known sexual dimorphism trends related to body weight and bone microarchitecture in mice (Maric et al., 2022; Rathod and Di Fulvio, 2021; Shim et al., 2022), male mice, regardless of genotype, had greater body weights (Fig. S12) and bone microarchitecture outcomes associated with greater bone mass in the femurs and L_4_ vertebrae compared to those of female mice (Table 1).

**Table 1.**
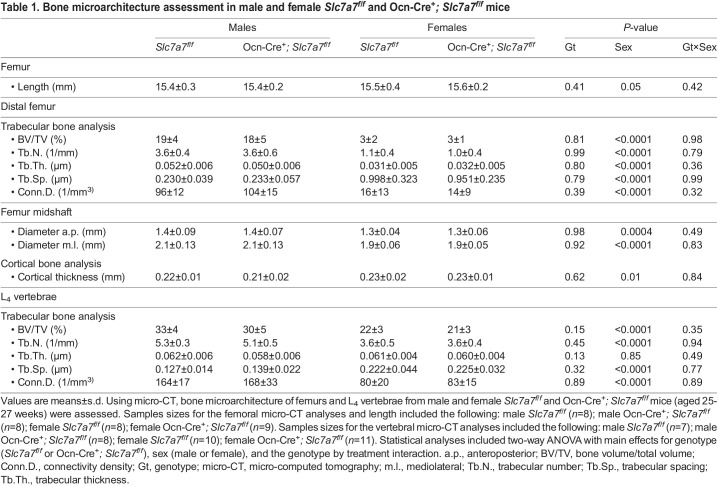
Bone microarchitecture assessment in male and female *Slc7a7^f/f^* and Ocn-Cre^+^*; Slc7a7^f/f^* mice

### Deletion of *Slc7a7* in osteo-chondroprogenitor cells does not promote skeletal defects

Given that Ocn-driven Cre transgene is expressed in mature osteoblasts, we next tested whether loss of *Slc7a7* earlier in osteoblast development impacts the skeletal phenotype. To this end, we used mice expressing Cre recombinase under the Prx1 promoter and enhancer (Logan et al., 2002). We similarly confirmed the activity of the Prx1-Cre transgene by crossing the mice with mice harboring the Rosa26 knock-in Cre reporter [Gt(ROSA)26Sortm6(CAG-ZsGreen1)Hze*;* Ai6]. As expected, we observed Ai6-expressing cells in the long bones, but not in the vertebrae or kidney (Fig. S13) or liver (Fig. S10) (Madisen et al., 2010). In addition, we observed a significant reduction in intact *Slc7a7* expression in the femurs of adult Prx1-Cre^+^*;* Slc7a7*^f/f^* versus *Slc7a7^f/f^* mice (Fig. S14).

At 2 months of age, Prx1-Cre^+^*; Slc7a7^f/f^* mice demonstrated similar growth to that of sex-matched *Slc7a7^f/f^* littermates (Fig. S15). We performed micro-CT in the femurs and vertebrae of male and female Prx1-Cre^+^*; Slc7a7^f/f^* and *Slc7a7^f/f^* mice. The femurs of Prx1-Cre^+^*; Slc7a7^f/f^* mice exhibited similar metrics related to size (i.e. length), in addition to cortical and trabecular architecture, to those of sex-matched *Slc7a7^f/f^* littermates (Table 2). Prx1-Cre is not expressed in the vertebrae, and, as expected, trabecular microarchitecture outcomes were also similar in the L_4_ vertebrae of Prx1-Cre^+^*; Slc7a7^f/f^* and *Slc7a7^f/f^* mice, regardless of sex (Table 2). The expected increases in body weight and bone mass in male versus female mice were observed (Table 2; Fig. S14).

**Table 2.**
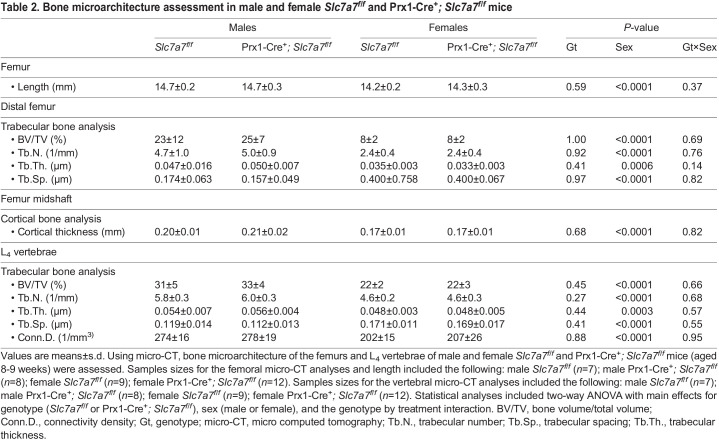
Bone microarchitecture assessment in male and female *Slc7a7^f/f^* and Prx1-Cre^+^*; Slc7a7^f/f^* mice

### Gene expression studies in the femurs/tibias and calvaria of *Slc7a7^Lbu/Lbu^* mice

**Fig. 6.**
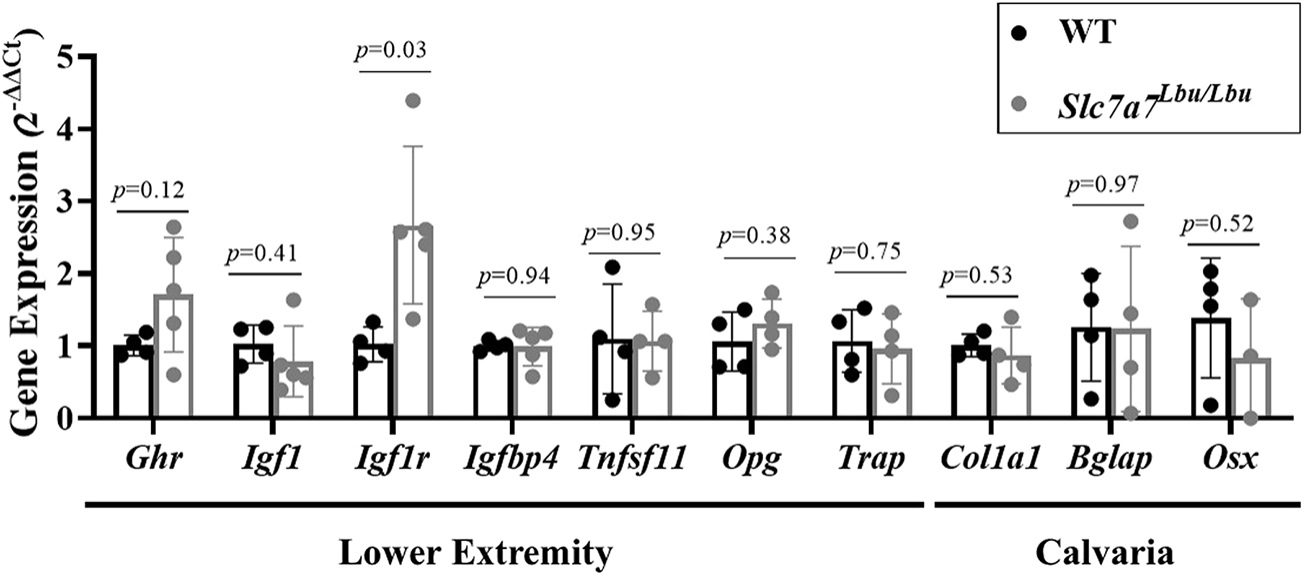
**Gene expression studies in the lower extremities and calvarias of WT versus *Slc7a7^Lbu/Lbu^* mice.** The lower extremities (femur/tibias were combined and bone marrow was flushed) and calvarias from WT and *Slc7a7^Lbu/Lbu^* mice were harvested at P14-P18. Gene expression was evaluated using quantitative PCR. Statistical analyses included unpaired two-tailed *t*-tests (*n*=4-5 per group). WT, wild type.

Owing to the surprising findings of normal growth and bone microarchitecture in the Ocn-Cre^+^*; Slc7a7^f/f^* mice and Prx1-Cre^+^*; Slc7a7^f/f^* mice, we utilized the *Slc7a7^Lbu/Lbu^* mouse model to test for alterations in the local expression of genes associated with osteoblast and osteoclast biology, in addition to the GH/IGF-1 axis, in combined femurs/tibias and in calvaria (Fig. 6). Although we observed a significant increase in the relative expression of *Igf1r* in the lower extremities of *Slc7a7^Lbu/Lbu^* mice, we observed similar expression of genes relevant to the GH/IGF-1 axis (*Ghr*, *Igf1*, *Igfbp4*) and osteoclast biology (*Tnfsf11*, *Tnfrsf11b*, *Trap*). We also observed similar expression of other genes relevant to osteoblast biology (*Col1a1*, *Bglap*, *Sp7*) in the calvarias of WT and *Slc7a7^Lbu/Lbu^* mice.

## DISCUSSION

LPI is an inborn error of metabolism for which the etiology of its diverse phenotypes involving multiple organ systems remains poorly understood (Ogier de Baulny et al., 2012; Contreras et al., 2021). The decreased availability of urea cycle intermediates (arginine and ornithine) due to impaired cationic amino acid transport likely contributes to phenotypes associated with urea cycle dysfunction, such as dietary protein aversion, hyperammonemia and neurological complications (Ogier de Baulny et al., 2012; Rajantie et al., 1980a, 1983a,b). However, urea cycle dysfunction is unlikely to be the sole contributor to the other phenotypes associated with LPI, such as failure to thrive, delayed bone age and osteoporosis, as these phenotypes are not typically observed in individuals with other disorders characterized by urea cycle dysfunction. Therefore, viable global and conditional knockout animal models that recapitulate features of human LPI are critical for investigations of tissue-specific pathological mechanisms and potential interventions. Hence, we developed a global *Slc7a7* knockout mouse model (*Slc7a7^Lbu/Lbu^*) and a conditional *Slc7a7* knockout mouse model (*Slc7a7^f/f^*) to further investigate aspects of growth failure, skeletal defects and osteoporosis associated with *Slc7a7* deficiency (Stroup et al., 2020).

We previously reported that our *Slc7a7^Lbu/Lbu^* mouse model (C57BL/6×129/SvEv F2) exhibits characteristic features of human LPI, including the classical biochemical phenotype, severe postnatal growth failure, proximal tubular dysfunction and delayed development in several organ systems (i.e. L_4_ vertebrae, lungs and kidney) (Stroup et al., 2020). In the present study, we demonstrated that the *Slc7a7^Lbu/Lbu^* embryos (C57BL/6×129/SvEv F2 background) exhibited 25% lower body weights than those of WT embryos at E17.5, which is consistent with the IUGR reported in three *Slc7a7*-deficient embryo models (Stroup et al., 2020; Sperandeo et al., 2007). Notably, despite severe postnatal growth failure (70% lower body weights), the *Slc7a7^Lbu/Lbu^* mouse model demonstrated mild IUGR, which indicates prenatal onset of growth failure that worsens during the postnatal period with global *Slc7a7* deficiency. This finding is consistent with multiple descriptions of human patients with LPI who exhibited low-normal growth during early infancy that worsened with age, particularly after the transition from low-protein breastmilk or infant formula to solid foods containing more dietary protein (Carpenter et al., 1985; Parini et al., 1991; Al-Qattan et al., 2021; No author listed, 1986; Maines et al., 2013). This difference in the severity of the prenatal versus postnatal growth failure may also suggest differences in the underlying pathological mechanisms. The idea that distinct mechanisms could be driving intrauterine and postnatal growth deficiencies in LPI is plausible given the differences in physiology and metabolism, including placental development and function required for intrauterine growth and differences in metabolism that could affect potent drivers of growth (i.e. GH/IGF-1 axis) (Poudel et al., 2020; Yakar and Isaksson, 2016).

First, we hypothesized that *Slc7a7*-deficient embryos may demonstrate IUGR partially due to alterations in the placenta, using placenta weight and histology as surrogate indicators of placental function. The hypothesis that reduced *SLC7A7* expression in placental tissue could hinder intrauterine growth is reasonable, given that reduced gene and protein expression of SLC7A7 in human fetal-derived chorionic tissue of the placenta was observed in infants with IUGR (Huang et al., 2018). Although the total weights of the placentas and embryos were 25% lower in *Slc7a7^Lbu/Lbu^* versus WT embryos (C57BL/6×129/SvEv F2), the placenta-to-embryo weight ratios were similar (Fig. 2). Furthermore, placenta microarchitecture appeared largely similar upon histologic examination (Fig. S1). Interestingly, a study in a fetal piglet model demonstrated increased expression of *SLC7A7* in chorionic placental tissue at mid-gestation when rapid fetal growth occurs (Krombeen et al., 2019). The authors speculated that increased *SLC7A7* expression in fetal piglet-derived chorionic placenta tissue at mid-gestation facilitates nutrient transport during this period of rapid fetal growth (Krombeen et al., 2019). Thus, it is unclear whether IUGR in this model is driven by impaired nutrient transport in the placenta, loss of *Slc7a7* in the embryo or both. Moreover, a human study reported that 21% of infants (four of 19) born to nine individuals with maternal LPI were small for gestational age despite normal placenta size (Tanner et al., 2006). Notably, only one of these 19 infants was reported to have LPI while the remainder were presumed to be heterozygous for a pathogenic variant in *SLC7A7* (Tanner et al., 2006). Because all nine mothers had LPI, it is likely that *SLC7A7* deficiency was present in the maternal decidua of the placenta for all 19 pregnancies; however, some *SLC7A7* expression was likely to be present in the fetal-derived chorionic placenta tissue for the majority of the pregnancies in this study (Tanner et al., 2006). Regardless, further studies that assess placental function and nutrient transport across gestation are needed to determine whether deficient amino acid transport across the placenta contributes to IUGR in *Slc7a7*-deficient embryos.

Second, we hypothesized that our global *Slc7a7^Lbu/Lbu^* mouse model would demonstrate similar plasma IGF-1 concentrations to those of WT controls prenatally but would exhibit IGF-1 deficiency postnatally. The GH/IGF-1 axis is a driver of somatic and longitudinal bone growth via endocrine signaling action as part of the somatotropic–hypothalamic–pituitary axis (Tsugawa et al., 2019; Oh et al., 2017; Poudel et al., 2020; Yakar and Isaksson, 2016). Pituitary-derived GH is the main regulator of hepatic IGF-1 production (Poudel et al., 2020; Yakar and Isaksson, 2016). In addition to hormones, a variety of factors, including arginine, are required for hepatic secretion of GH and IGF-1 into the circulation (Tsugawa et al., 2019; Oh et al., 2017; Poudel et al., 2020; Yakar and Isaksson, 2016). The majority of serum IGF-1 is synthesized in the liver (75-80%), while the remaining 20-25% of circulating IGF-1 is derived from other tissues (primarily muscle and adipose tissue) (Yakar and Isaksson, 2016; Kawai and Rosen, 2012). Activation of the IGF-1 receptor (IGF1R) by IGF-1 triggers downstream signaling cascades involving phosphatidylinositol 3-kinase (PI3K) and mitogen-activated protein kinase (MAPK) for cell survival, hypertrophy and proliferation in many tissues, including the liver, adipose tissue, muscle and bone (Poudel et al., 2020). Many hypothesize that GH/IGF-1 axis disturbances are a major contributor to the growth failure in LPI owing to the relationship between growth hormone insensitivity syndromes and short stature (Andrews et al., 2021), in addition to the numerous reports of low concentrations of circulating GH or IGF-1 in children with LPI that exhibit growth failure (Goto et al., 1984; Esposito et al., 2006; Evelina et al., 2015; Al-Qattan et al., 2021; Niinikoski et al., 2011; Cottrell and Mushtaq, 2016). Consistent with these human studies, we observed 87% lower plasma IGF-1 concentrations in *Slc7a7^Lbu/Lbu^* mice compared to those in WT mice (aged P14-P18) (Fig. 3B), which complements our previous report describing the plasma arginine deficiency and reduced hepatic expression of *Igf1* in this model (Stroup et al., 2020). Plasma IGF-1 concentrations were similar in *Slc7a7^Lbu/Lbu^* and WT embryos (aged E17.5), which was not surprising as we predict that *Slc7a7^Lbu/Lbu^* embryos likely have sufficient circulating arginine. Although hepatic expression of *Igf1* was not assessed in the *Slc7a7^Lbu/Lbu^* embryos, reduced hepatic expression of *Igf1* in *Slc7a7^−/−^* embryos has been reported (Sperandeo et al., 2007). Collectively, these findings may indicate a postnatal onset of systemic IGF-1 deficiency in *Slc7a7*-deficient mice. Further studies are needed to address whether this IGF-1 deficiency is due to nutritional factors, such as low circulating arginine, or whether other factors contribute to this finding.

The presence of systemic IGF-1 deficiency and more severe growth deficits at the postnatal time point (P14-P18) in our *Slc7a7^Lbu/Lbu^* mouse model may signify that IGF-1 deficiency plays a larger role during postnatal growth, rather than embryonic growth, in the setting of global *Slc7a7* deficiency. Similar to the poor survival and growth deficits in the *Slc7a7*-deficient mouse models (Stroup et al., 2020; Sperandeo et al., 2007; Bodoy et al., 2019), global *Igf1^−/−^* and *Igf1r^−/−^* mouse models with depleted circulating IGF-1 concentrations manifested early lethality (due to respiratory failure) and growth failure (27-60% of WT body weights) (Liu et al., 1993; Powell-Braxton et al., 1993; Wang et al., 2006; Mohan et al., 2003). Although IGF-1 is critical for embryonic growth and skeletal development (Liu et al., 1993; Wang et al., 2006), the growth deficits in the *Igf1-* or *Igf1r-*deficient mice as a percentage of their WT controls appeared more severe during postnatal development, and the gaps between the mutant and WT growth curves widened with age (Powell-Braxton et al., 1993; Mohan et al., 2003; Baker et al., 1993). In addition to the early lethality and growth failure observed in the *Igf1-* or *Igf1r-*deficient mouse models, delayed skeletal development with delays in overall ossification or the formation of the ossification centers of the long bones were described (Liu et al., 1993; Wang et al., 2006; Mohan et al., 2003). Consistent with the delayed skeletal development observed in *Igf1-* or *Igf1r-*deficient mice (Liu et al., 1993; Wang et al., 2006; Mohan et al., 2003), *Slc7a7^Lbu/Lbu^* mice also exhibited delayed skeletal development as evidenced by smaller or absent secondary ossification centers in the femurs and tibias, in addition to the retention of the vertebral growth plates within the spine (Figs 4 and 5; Figs S2 and S3). Taken together, perturbations in the GH/IGF-1 axis may contribute to the growth failure and delayed skeletal development phenotypes in *Slc7a7^Lbu/Lbu^* mice.

In contrast to the severe deficits in growth and skeletal development in global *Igf1* and *Igf1r* knockout mouse models (Liu et al., 1993; Wang et al., 2006; Mohan et al., 2003), liver-specific knockout mouse models that disrupt the endocrine action of IGF-1 exhibited no change or mild reductions in body weight (0-30% lower) and long bone length (0-10% lower) compared to controls (Yakar and Isaksson, 2016; Nordstrom et al., 2011; Courtland et al., 2010; Sjogren et al., 1999, 2002). Similar to the liver-specific knockout mouse models with low serum IGF-1 concentrations, mouse models with conditional deletion of *Igf1* or *Igf1r* in osteoblasts using Ocn-Cre (Zhang et al., 2002; Wang et al., 2007) or Col1a2-Cre (Govoni et al., 2007b; Kesavan et al., 2011), or chondrocytes using Col2a1-Cre, also revealed mild reductions in body weight (10-30% lower) and long bone length (3-25% lower) (Govoni et al., 2007a; Wang et al., 2011; Wu et al., 2015). Overall, these global and tissue-specific *Igf1* and *Igf1r* knockout mouse models illustrate both the individual and synergistic impact of endocrine and local IGF-1 action for growth and skeletal development.

Recently, an adult *Slc7a7*-deficient mouse model was generated using a conditional allele in the setting of a globally expressed, tamoxifen-inducible Cre recombinase (UBC-Cre-ERT2^+^*; Slc7a7^f/f^*) (Bodoy et al., 2019; Giroud-Gerbetant et al., 2022 preprint). This adult inducible mouse model recapitulates aspects of the human disorder, including the biochemical phenotype with reduced plasma concentrations and impaired renal reabsorption of the cationic amino acids, urea cycle dysfunction with hyperammonemia and hyperexcretion of urinary orotic acid, and pulmonary alveolar proteinosis with pulmonary fibrosis and alveolar surfactant protein-B accumulation (Bodoy et al., 2019). However, a low-protein diet and L-citrulline supplementation were required to attenuate survival and weight loss. Nonetheless, potential mechanisms contributing to growth failure and delayed skeletal development, common complications of LPI (Svedstrom et al., 1993; Contreras et al., 2021), were not addressed in these studies using this conditional *Slc7a7* knockout mouse model (Bodoy et al., 2019; Giroud-Gerbetant et al., 2022 preprint).

To test the hypothesis that *Slc7a7* deficiency in mature osteoblasts or mesenchymal cells of the osteo-chondroprogenitor lineage hinders growth and skeletal development and leads to osteoporosis *in vivo*, we generated a conditional *Slc7a7* mouse model and deleted *Slc7a7* in the mature osteoblasts using the Ocn-Cre and in the osteo-chondroprogenitor cells using Prx1-Cre (Figs S8-S15). We developed our hypothesis based on the following preclinical data: (1) growth failure and delayed skeletal development in the setting of IGF-1 deficiency in *Slc7a7^Lbu/Lbu^* mice (Figs 4 and 5); (2) reduced osteoblast differentiation and mineralization reported in *Slc7a7*-deficient ST2-cell derived osteoblasts (Shen et al., 2021); (3) local production and secretion of IGF-1 by osteoprogenitors and osteoblasts for endochondral ossification and endosteal bone formation (Yakar and Isaksson, 2016; Wang et al., 2006); and (4) growth and skeletal defects reported in the osteoblast-specific *Igf1r* knockout mouse models using Ocn-Cre (Zhang et al., 2002; Wang et al., 2007). However, based on micro-CT, the trabecular architecture in the L_4_ vertebrae and the trabecular and cortical architecture in the distal femurs were similar in male and female Ocn-Cre^+^*; Slc7a7^f/f^* mice compared to sex-matched controls (Table 1). Similar results were obtained with the male and female Prx1-Cre^+^*; Slc7a7^f/f^* mice compared to sex-matched controls (Table 2).

Furthermore, growth curves from 4 to 24 weeks of age were similar in male and female Ocn-Cre^+^*; Slc7a7^f/f^* mice compared to sex-matched controls, and in the male and female Prx1-Cre^+^; *Slc7a7^f/f^* mice compared to sex-matched controls from 4 to 8 weeks of age (Figs S12 and S15). Despite the reduced von Kossa or alkaline phosphatase staining suggestive of impaired osteoblast mineralization or differentiation, respectively, in *Slc7a7*-deficient ST2 cell-derived osteoblasts (Shen et al., 2021), our mouse studies suggest that conditional deletion of *Slc7a7* in osteo-chondroprogenitor cells or mature osteoblasts alone may not have a significant impact on bone formation *in vivo*.

Owing to the normal growth and bone architecture in the Ocn-Cre^+^*; Slc7a7^f/f^* and Prx1-Cre^+^*; Slc7a7^f/f^* mice and the contrasting strong phenotype in the *Slc7a7^Lbu/Lbu^* mice, we assessed the expression of genes relevant to osteoblasts, osteoclasts and the GH/IGF-1 axis in the *Slc7a7^Lbu/Lbu^* mice (Fig. 6). No major disruptions in the expression of osteoblast markers in calvaria or expression of osteoclast markers or GH/IGF-1 axis genes in the shafts of combined femurs/tibias were observed in the *Slc7a7^Lbu/Lbu^* mice. However, osteoprogenitors and chondrocytes in the growth plate regions of the long bones express *Igf1* and other GH/IGF-1 axis genes (Yakar and Isaksson, 2016; Wang et al., 2006). Moreover, multiple cell types are present in these tissues and, thus, small changes in gene expression in particular cell types, such as osteoclasts and chondrocytes, may not be detectable in our gene expression studies. Thus, further studies targeting the expression of GH/IGF-1 axis genes in the growth plate regions of the long bones are needed before conclusions can be made regarding potential alterations in spatio-temporal expression.

Limitations of this work included the preweaning lethality and growth failure of the *Slc7a7^Lbu/Lbu^* mice, which confined our investigations to tissue harvests through ∼18 days of age (Stroup et al., 2020). Consequently, we were unable to utilize our *Slc7a7^Lbu/Lbu^* mouse model to assess phenotypes in skeletally mature mice or pursue intervention studies. For example, we attempted a pilot intervention study to rescue the systemic IGF-1 deficiency with intraperitoneal injections containing recombinant human IGF-1 (1 mg/kg) or a saline control every 48 h from P5 through P15. However, our pilot intervention studies could not be optimized or interpreted owing to the fragility of the surviving *Slc7a7^Lbu/Lbu^* mice. Moreover, amino acid metabolism has an important role in osteoblast differentiation (Shen et al., 2022), and it is possible that the skeletal phenotype is driven by osteoblast dysfunction caused by deficiency of arginine, lysine and ornithine in LPI. However, as this amino acid deficiency is caused by loss of *Slc7a7* in intestinal epithelial cells and renal tubular cells, the mice with osteoblast-specific and osteo-chondroprogenitor cell-specific loss of *Slc7a7* would not be expected to demonstrate a phenotype if this is the case. Lastly, our results must be interpreted in the context of the fact that tissue-specific Cre recombinases are not typically 100% efficient, which could impact the findings in studies using these models.

In summary, we demonstrated that *Slc7a7^Lbu/Lbu^* mice exhibited severe postnatal growth failure and delayed skeletal development in the setting of systemic IGF-1 deficiency. In contrast to the severe growth failure and systemic IGF-1 deficiency in the *Slc7a7^Lbu/Lbu^* mice, we observed mild IUGR in *Slc7a7^Lbu/Lbu^* mouse embryos despite normal circulating IGF-1 concentrations, which might suggest different underlying mechanisms driving growth failure in embryonic versus postnatal development. To overcome the preweaning lethality and severe growth failure in the *Slc7a7^Lbu/Lbu^* mouse model, we generated an adult conditional *Slc7a7* knockout mouse model. Our Prx1-Cre^+^*; Slc7a7^f/f^* and Ocn-Cre^+^*; Slc7a7^f/f^* mouse models suggested that *Slc7a7* deficiency in the osteoblastic lineage may not be a major contributor to the growth or skeletal phenotypes associated with LPI. However, *Slc7a7* is expressed in other bone cell types, such as osteoclasts and osteoclast precursors (Madel et al., 2020; Tsukasaki et al., 2020), and thus additional conditional *Slc7a7* knockout mouse models that target other skeletal cell types are needed to evaluate the role of *Slc7a7* in other bone cells. Alternatively, it is possible that a non-cell-autonomous mechanism, such as deficiency of circulating arginine, ornithine and/or lysine or perturbations in circulating immune factors, may contribute to the growth failure and/or skeletal defects in the *Slc7a7^Lbu/Lbu^* mouse model. Regardless, future *in vivo* studies utilizing tissue-specific and developmental stage-specific *Slc7a7* knockout mouse models will advance our understanding of potential cell-autonomous or non-cell-autonomous mechanisms underlying growth failure, delayed skeletal development and osteoporosis in LPI.

## MATERIALS AND METHODS

### Generation of *Slc7a7*-deficient mouse models

The generation of the global *Slc7a7* knockout mouse model on the C57BL/6×129/SvEv F2 background used in these studies was previously described, and the colony was maintained by intercrossing *Slc7a7* heterozygous F1 mice using a trio breeding scheme (Stroup et al., 2020).

To generate *Slc7a7* conditional knockout mice (*Slc7a7^f/f^*), a C57BL/6N JM8A3.N1 embryonic stem cell clone (EPD0803_3_C07) harboring the tm1a(EUCOMM)Wtsi knockout first allele (Fig. S5A) was obtained from the European Mutant Mouse Cell Repository (EUMMCR). Allele quality control was performed using standard PCR to confirm the presence of the following sequences: loxP 3′ of exon 4 of *Slc7a7*, lacZ and Neo (Fig. S5B). Quantitative TaqMan-based PCR copy number counting for lacZ (Thermo Fisher Scientific, 4400291, Assay ID Mr00529369_cn) and the loxP-flanked *Slc7a7* sequence (Thermo Fisher Scientific, 4400291, Assay ID Mm00413101_cn) was then used to exclude random integration or multiple copies of the target construct in the genome (Fig. S5C,D). Genomic DNA from an established mouse line [C57BL/6N-*Prdm14^tm1a(EUCOMM)Wtsi^*] was used to generate the WT, heterozygous and homozygous copy number PCR controls. A TaqMan assay for *Tfrc* was used as a copy number control (Thermo Fisher Scientific, 4458366). Following the allele quality control experiments, murine blastocysts (albino C57BL/6 background) were injected with embryonic stem cells, using standard conditions, to generate the chimeras. The chimeras were bred to C57BL/6NTac-*Tyr^tm1Arte^* mice and allele transmission was confirmed by TaqMan PCR for the lacZ sequence. To convert the tm1a knockout first allele to a tm1c(EUCOMM)Wtsi conditional knockout allele, C57BL/6N-*Slc7a7^tm1a(EUCOMM)Wtsi^* mice were bred to B6.129S4-Gt(ROSA)26Sor^tm1(FLP1)Dym/^RainJ mice (The Jackson Laboratory, 009086) to remove the Flippase recognition target-flanked lacZ/Neo cassette (Fig. S5A). The tm1c allele was maintained using backcrosses to C57BL/6 mice.

Ocn-Cre mice [B6.FVB-Tg(BGLAP-cre)1Clem/J; The Jackson Laboratory, 019509] were crossed with mice harboring the *Slc7a7* conditional allele (Zhang et al., 2002). To generate the male and female Ocn-Cre*^+^; Slc7a7^f/f^* and *Slc7a7^f/f^* mice used for experiments, female *Slc7a7^f/f^* mice were crossed with male Ocn-Cre^+^; *Slc7a7^f/+^*mice using a pair breeding scheme. Prx1-Cre mice [B6.Cg-Tg(Prrx1-cre)1Cjt/J; The Jackson Laboratory, 005584) were crossed with mice harboring the *Slc7a7* conditional allele (Logan et al., 2002). To generate the male and female Prx1-Cre*^+^; Slc7a7^f/f^* and *Slc7a7^f/f^* mice used for experiments, female *Slc7a7^f/f^* mice were crossed with male Prx1-Cre^+^*; Slc7a7^f/+^*mice. Primers and product sizes for standard PCR reactions for the generation and maintenance of the *Slc7a7^f/f^*, Ocn-Cre^+^*; Slc7a7^f/f^* and Prx1-Cre^+^*; Slc7a7^f/f^* mice can be found in Table S2.

### Mouse colony management

All mouse colonies were housed in the Baylor College of Medicine Transgenic Mouse Facility. The mice were maintained in microisolator cages with ventilated racks connected to an automated autoclaved water system with free access. The room in which the mice were housed maintained a standard 12 h light/12 h dark cycle. Mice had *ad libitum* access to an extruded chow diet (LabDiet 5V5R formulation). Because the WT and *Slc7a7^Lbu/Lbu^* mice (aged P14-P18) were dissected prior to weaning, we predict that these mice primarily consumed breast milk. Cre^+^*; Slc7a7^f/f^* and *Slc7a7^f/f^* mice were weaned at 21 days of age. Male and female mice were used for all studies unless otherwise specified. All studies were approved by the Baylor College of Medicine Institutional Animal Care and Use Committee. All primers used for genotyping are listed in Table S1.

### Embryo and placenta assessments

To generate WT and *Slc7a7^Lbu/Lbu^* embryos, a standard timed mating approach was used. Specifically, using a pair breeding scheme, female F1 *Slc7a7^Lbu/+^* mice were intercrossed with male F1 *Slc7a7^Lbu/+^* mice. We checked for vaginal plugs in the mornings, and the male F1 *Slc7a7^Lbu/+^* mice were removed when a vaginal plug was observed (E0.5). Embryos and their placentas were harvested and weighed on E17.5. Placentas were fixed in cold 4% paraformaldehyde for 48 h and then washed in 1× phosphate buffered saline and stored in 70% ethanol at 4°C. Placentas were processed, paraffin embedded, sectioned (7 µm thickness), stained with H&E using standard protocols and reviewed by a pathologist.

### Primary calvaria-derived osteoblast cultures for *Slc7a7^Lbu/Lbu^* mouse studies

Calvaria were dissected from E18.5 *Slc7a7^Lbu/Lbu^* pups and littermate controls. Each calvaria was dissected by incubation in digestion medium, composed of alpha-minimum essential medium (MEM) with 0.05% Trypsin-EDTA and 0.1 mg/ml collagenase P (Sigma-Aldrich) for 2.5 h with frequent shaking and pipetting. Cultures were then incubated in enriched growth medium, composed of alpha-MEM containing 15% fetal bovine serum (FBS), 1% glutamine and 1% penicillin/streptomycin (P/S) in six-well plates for 5 days, until confluency was achieved. On the fifth day, cells were reseeded into 12-well plates at 1×10^5^ cells/ml in differentiation medium, composed of alpha-MEM with 10% FBS, 1% glutamine, 1% P/S, 10 mM beta glycerophosphate (Sigma-Aldrich) and 100 µg/ml ascorbic acid (Sigma-Aldrich). Cells were cultured in differentiation medium for 7 days to measure phosphatase activity, or 15 days to assess mineralization, then fixed and stained as specified below.

### Mineralization assessment and staining for alkaline phosphatase activity

Primary calvaria cells from WT and *Slc7a7^Lbu/Lbu^* embryos were fixed in 4% paraformaldehyde in PBS for 20 min. For measurement of alkaline phosphatase activity, fixed cultures were incubated for 20 min in staining solution, containing Naphthol AS-MX phosphate (0.1 mg/ml), N,N dimethyl formamide (0.5%), MgCl_2_ (2 mM), Fast Blue BB salt (0.6 mg/ml) and Tris-HCl pH 8.5 (0.1 M). For mineralization assessment, fixed cultures were incubated for 20 min in Alizarin Red solution (40 mM). Stained cultures were washed in PBS and dried for scanning.

### Plasma IGF-1 measurement

Peripheral blood was collected via retro-orbital bleeding from WT and *Slc7a7^Lbu/Lbu^* mice at P14-P18 or trunk blood was collected from decapitated WT and *Slc7a7^Lbu/Lbu^* embryos at E17.5. Regardless of the blood collection method, the blood was collected into lithium heparinized tubes and centrifuged (2000 ***g*** for 20 min at 4°C), after which the plasma was transferred into 1.5 ml microcentrifuge tubes. Aliquots of plasma were snap frozen using liquid nitrogen and stored at −20°C. To assess plasma concentrations of IGF-1, we performed a solid-phase sandwich enzyme-linked immunoassay in duplicate according to the manufacturer's instructions (R&D Systems, MG100). Optical density was assessed using a Tecan Infinite M200 Pro plater reader (Tecan Group Ltd, Männedorf, Switzerland) set to 450 nm with a wavelength correction of 540 nm.

### Skeletal histology and radiography

Lower extremities (femurs and tibias left attached) and spines from P14-P18 WT and *Slc7a7^Lbu/Lbu^* mice were fixed in buffered formalin for 48 h and then washed in 1× phosphate buffered saline and stored in 70% ethanol at 4°C. These calcified lower extremities and spines were processed, embedded in methacrylate, sectioned (6 µm thickness), and stained with von Kossa or tartrate-resistant acid phosphatase (TRAP) using standard procedures. Skeletal radiography was performed with an XPERT 80 system (Kubtec Medical Imaging, Milford, CT, USA).

### Micro-CT imaging

Femurs and spines were harvested from male and female Ocn-Cre^+^*; Slc7a7^f/f^* and *Slc7a7^f/f^* mice (aged 25-27 weeks) and from male and female Prx1-Cre^+^*; Slc7a7^f/f^* and *Slc7a7^f/f^* mice (aged 8-9 weeks). The tissues were fixed in buffered formalin for 48 h and then washed in 1× phosphate buffered saline and stored in 70% ethanol at 4°C. The micro-CT imaging was performed with a Scanco µCT 40 System with 50-peak kV and 145 µA X-ray source (Scanco Medical AG, Bruttisellen, Switzerland). The L_4_ vertebrae were scanned at a 16 µm resolution for quantification of the skeletal parameters. The micro-CT analyses were performed using Scanco Medical AG software version 6.1, and investigators performed the analysis unaware of genotype. All analyses were performed with a lower threshold value of 250, and the noise filter was set to Gauss Sigma=0, Gauss Support=1. For the trabecular bone analyses of the L_4_ vertebrae, we analyzed the maximum number of slices for each vertebral body. Because we predicted differences in growth and, thus, femur length in the Cre^+^*; Slc7a7^f/f^* mouse model, the number of analyzed slices for the trabecular and cortical bone analyses in the femur were adjusted for length. We measured femur length from the top of the femoral head to the bottom of the medial condyle. For the trabecular bone analyses of the femurs, we analyzed five slices per mm of femur length (74-80 slices or 1.18-1.28 mm of cancellous bone) at the distal metaphysis. For the cortical bone analyses of the femurs, we analyzed three slices per mm of femur length (45-49 slices or 0.72-0.78 mm) at the femoral midshaft.

### Immunofluorescence studies to assess Cre activity

We crossed female mice harboring the Ai6 Cre reporter transgene [B6.Cg-*Gt(ROSA)26Sor ^tm6(CAG-ZsGreen1)Hze^*/J; The Jackson Laboratory, 007906] with male mice harboring the Ocn-Cre or Prx1-Cre transgene. Pups with the Ai6^+^ (control) or Ai6^+^*;* Cre^+^ genotypes were decapitated and dissected for tissue harvest at 1 day of age. The decapitated heads, liver, kidney and lower extremities were fixed in cold 4% paraformaldehyde for ∼1.5 h at 4°C. Tissues were washed with cold 1× phosphate buffered saline and transferred to a cold 30% sucrose solution for 48 h at 4°C. Prior to embedding, tissues were bathed in O.C.T. compound (Sakura, Tissue-Tek, 4583) for 30 min. Tissues were embedded in O.C.T. compound and stored at −80°C to harden. Tissues were cryosectioned and stained with 4′-6-diamidino-2-phenylindole (DAPI) using standard procedures. The fluorescent sections were visualized using an Axioscan.Z1 slide scanner (Carl Zeiss AG, Oberkochen, Germany).

### Primary calvaria-derived osteoblast cultures for Ocn-Cre^+^*; Slc7a7^f/f^* mouse studies

The calvarias from Ocn-Cre^+^*; Slc7a7^f/f^* and *Slc7a7^f/f^* pups (aged 3-5 days) were harvested, cleaned and placed in ice cold Dulbecco's phosphate buffered saline (DPBS) within a six-well plate on ice. The calvarias were transferred to a fresh well within a six-well plate containing 1 ml of digestion medium [75% Hyclone alpha-MEM (Cytiva, SH30265.01), 20% trypsin EDTA (0.25%) and 5% collagenase P (2 mg/ml, Sigma-Aldrich, 11213857001)] at room temperature. The calvarias were carefully split into pieces using a 1 ml pipette tip and shaken side-to-side. The plate was incubated for 30 min in an incubator (37°C, 5% carbon dioxide, ambient oxygen). The digestion medium was removed, and 1 ml digestion medium with 50 µl collagenase P (2 mg/ml) was added. The calvaria tissues were further broken up using a scalpel. The plate was incubated for 2 h with manual agitation every 10 min. After the 2 h incubation, we added 3.75 ml alpha-MEM complete medium [10% FBS, 1% P/S, 1% L-glutamine] with additional FBS to bring the final FBS concentration to 15%. The plate was stored in the incubator overnight.

The alpha-MEM complete medium was changed every 48 h. Confluence (70-80%) was achieved within 4 days, at which time the calvaria cells were re-seeded into a 12-well plate at a seeding density of 1×10^5^ cells per well using osteogenic medium to promote osteoblast differentiation. The osteogenic medium was comprised of alpha-MEM, 10% FBS, 1% P/S, 50 µg/ml ascorbic acid (Sigma-Aldrich, 255564-5G) and 10 mM beta-glycerol phosphate disodium salt hydrate (Sigma-Aldrich, G9422-50G). The osteogenic medium was changed every 1-2 days. Calvaria-derived osteoblasts received four treatments of osteogenic media and were cultured for ∼7 days.

Cell pellets were collected, snap frozen in liquid nitrogen and stored at −80°C. To extract genomic DNA from the cell pellet, a standard phenol–chloroform method was used. DNA copy number of exon 3 of *Slc7a7* was assessed in triplicate using a LightCycler^®^ 96 System (Roche Holding AG, Basel, Switzerland) with FastStart Essential DNA Master reagent (Roche Diagnostics, 06402712001). Primer sequences to assess relative DNA copy number of exon 3 of *Slc7a7* included the following: Forward, 5′-TGTTAGACTTGGCCAGGGTAAGAAA-′3; Reverse, 5′-CGGTGAAGGTGTAGATCTGAGACAC-′3.

### Quantitative real-time PCR for gene expression

For gene expression studies in the *Slc7a7^Lbu/Lbu^* mouse model, all tissues were harvested at 14-18 days of age. The calvaria was harvested, cleaned, fixed in TriZOL, and then snap frozen in liquid nitrogen and stored at −80°C. Total RNA was extracted from the TriZOL-fixed calvaria (Sigma-Aldrich, GenElute Mammalian Total RNA Miniprep Kit, RTN70-1KT). The femurs and tibias were also harvested and cleaned. The proximal and distal ends of the femurs and tibias were cut to flush the bone marrow using centrifugation (21,130 ***g***, 1 min, 4°C). The femurs and tibias were combined in a 1.5 ml microcentrifuge, snap frozen in liquid nitrogen and stored at −80°C. The femurs/tibias were later fixed in TriZOL, and total RNA was extracted using an alternative method (Qiagen, RNeasy Mini Kit, 74104; Qiagen, RNase-Free DNase Set, 79254).

For gene expression studies in the Ocn-Cre^+^*; Slc7a7^f/f^* and *Slc7a7^f/f^* mice, tissues were harvested at 25-27 weeks of age, and for gene expression studies in the Prx1-Cre^+^*; Slc7a7^f/f^* and *Slc7a7^f/f^* mice, the tissues were harvested at 8-9 weeks of age. The femurs were harvested and cleaned. The proximal and distal ends of the femur were cut to flush the bone marrow using centrifugation (21,130 ***g***, 1 min, 4°C). The femurs were then snap frozen in liquid nitrogen and stored at −80°C until RNA extraction. To extract total RNA from the femur, the femur was first homogenized using a T-25 digital ULTRA-TURRAX^®^ instrument (IKA Works GmbH & Co., Staufen, Germany) at maximal speed in TriZOL reagent. Total RNA was purified using a Direct-zol™ RNA MiniPrep Kit (Zymo Research, R2050). The quality and the integrity of the RNA was assessed by measuring optical density using a DS-11+ spectrophotometer (DeNovix) and gel electrophoresis using 1% agarose gel containing ethidium bromide in Tris-acetic acid-EDTA (TAE) buffer.

All total RNA was reversed transcribed using a Superscript III Synthesis System (Invitrogen, 18080051) with random hexamers. For the Ocn-Cre studies, real-time PCR was performed in triplicate using a LightCycler^®^ 96 System (Roche Holding AG, Basel, Switzerland) with FastStart Essential DNA Master Reagent (Roche Diagnostics, 06402712001). The primer sequences used to assess gene expression can be found in Table S2. Using *B2m* as the housekeeping gene, fold change in relative gene expression was estimated with the 2^-ΔΔCt^ method (Livak and Schmittgen, 2001). For Prx1-Cre, real-time PCR was performed in triplicate using a QuantStudio 7 Flex system (Applied Biosystems) with PowerUp SYBR Green Master Mix (Applied Biosystems, A25742). Non-template controls containing water instead of cDNA were included for each primer pair. The primer sequences used to assess gene expression can be found in Table S2. Using *Rpl7* as the housekeeping gene, fold change in relative gene expression was estimated with the 2^-ΔΔCt^ method.

### Statistical analysis

Two-way ANOVA using PROC GLM (SAS 9.4, SAS Institute Inc., Cary, NC, USA) was utilized to examine the body weights of male and female WT and *Slc7a7^Lbu/Lbu^* mice prior to dissections with main effects for genotype (WT or *Slc7a7^Lbu/Lbu^*) and sex (male or female) and the genotype by sex interaction. We performed statistical tests to determine whether the assumptions of normality and equal variance were met. If the data were skewed, a log transformation was performed. If log-transformed data remained skewed, we performed a two-way ANOVA on ranks using PROC RANK within PROC GLM (SAS Institute Inc.). A similar approach was used to assess micro-CT parameters in the Cre^+^*; Slc7a7^f/f^* mouse models. Two-way ANOVA with repeated measures (SAS) was used to assess the growth curves in male and female *Slc7a7^f/f^* and Cre^+^*; Slc7a7^f/f^* mice with main effects for genotype and sex and the genotype by sex interaction.

Unpaired two-tailed *t*-tests using PROC TTEST (SAS) or GraphPad Prism (version 9.4.1) were performed to investigate differences due to genotype for embryo body weights, placenta weights, embryo:placenta weight ratios, plasma IGF-1 concentrations, gene expression studies and DNA copy number in primary calvaria-derived osteoblast cultures. When data did not satisfy assumptions of normality, the Kruskal–Wallis test was performed. Because statistical assumptions were met, the raw data were used. Statistical significance was set at *P*<0.05.

## Supplementary Material

10.1242/dmm.050118_sup1 Supplementary information
