# Supplementary material for: Delayed skeletal development and IGF-1 deficiency in a mouse model of lysinuric protein intolerance
Source: Dis Model Mech. 2023 Aug 17;16(8):dmm050118. doi: 10.1242/dmm.050118 (PMC10445726; doi:10.1242/dmm.050118)
Supplement: Supplementary information [file dmm-16-050118-s1.pdf]

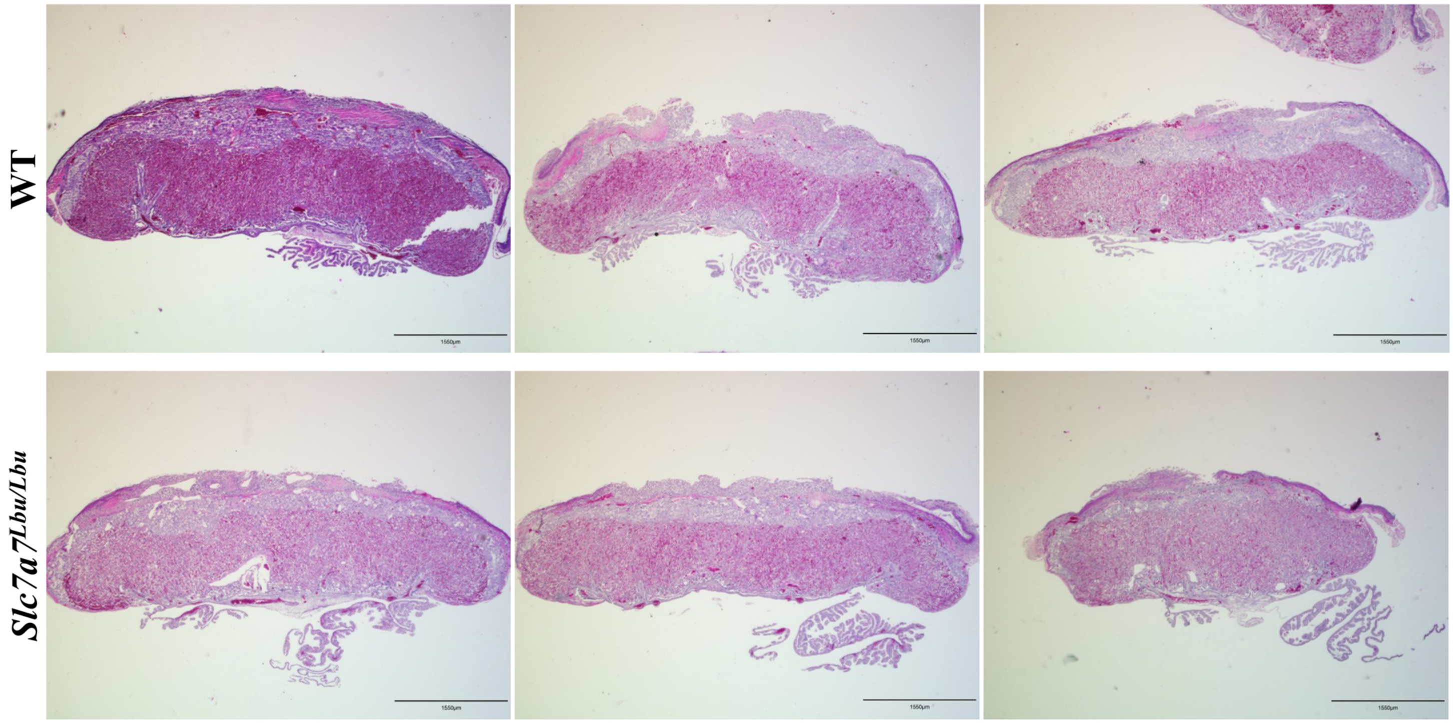

Fig. S1. Microarchitecture of the placentas from WT and *Slc7a7*$^{Lbu/Lbu}$ embryos. H&E- stained placenta sections from WT and *Slc7a7*$^{Lbu/Lbu}$ embryos at E17.5. H&E, hematoxylin and eosin; WT, wild type.

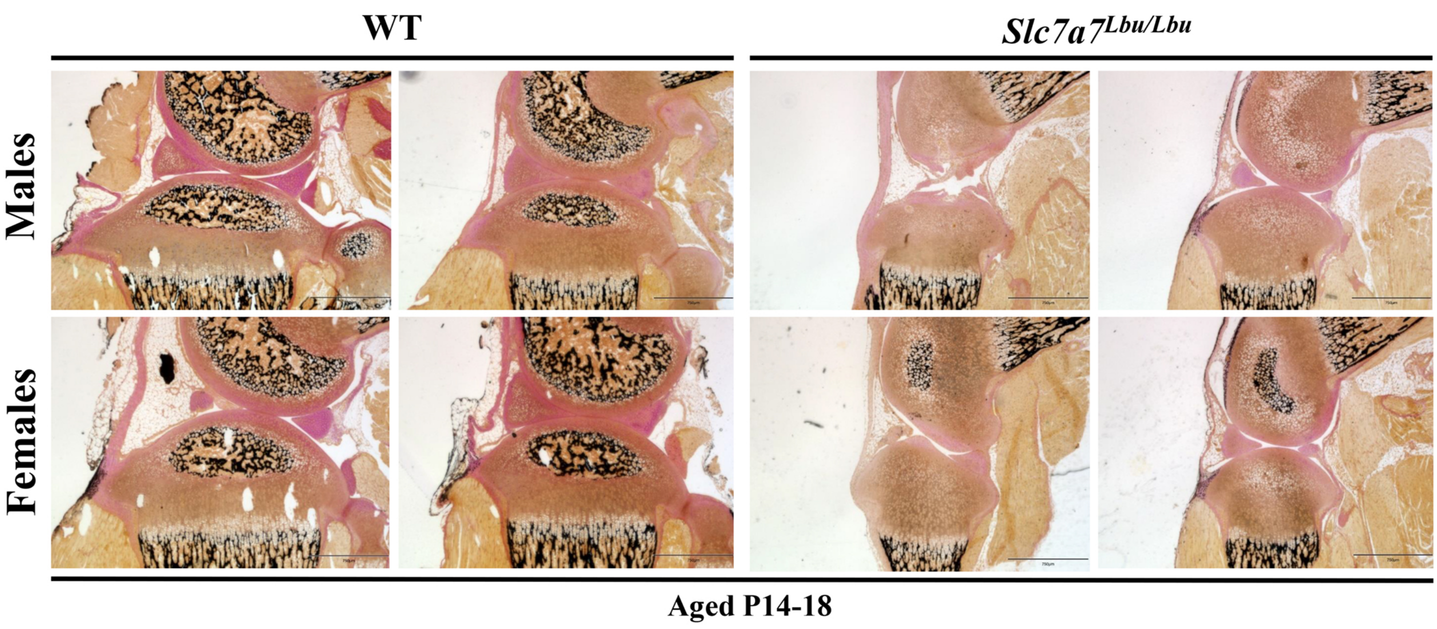

Fig. S2. Variability in the histological architecture of von Kossa-stained lower extremity sections in WT and *Slc7a7*$^{Lbu/Lbu}$ mice at P14-18. Scale bars indicate 750 µm. WT, wild type.

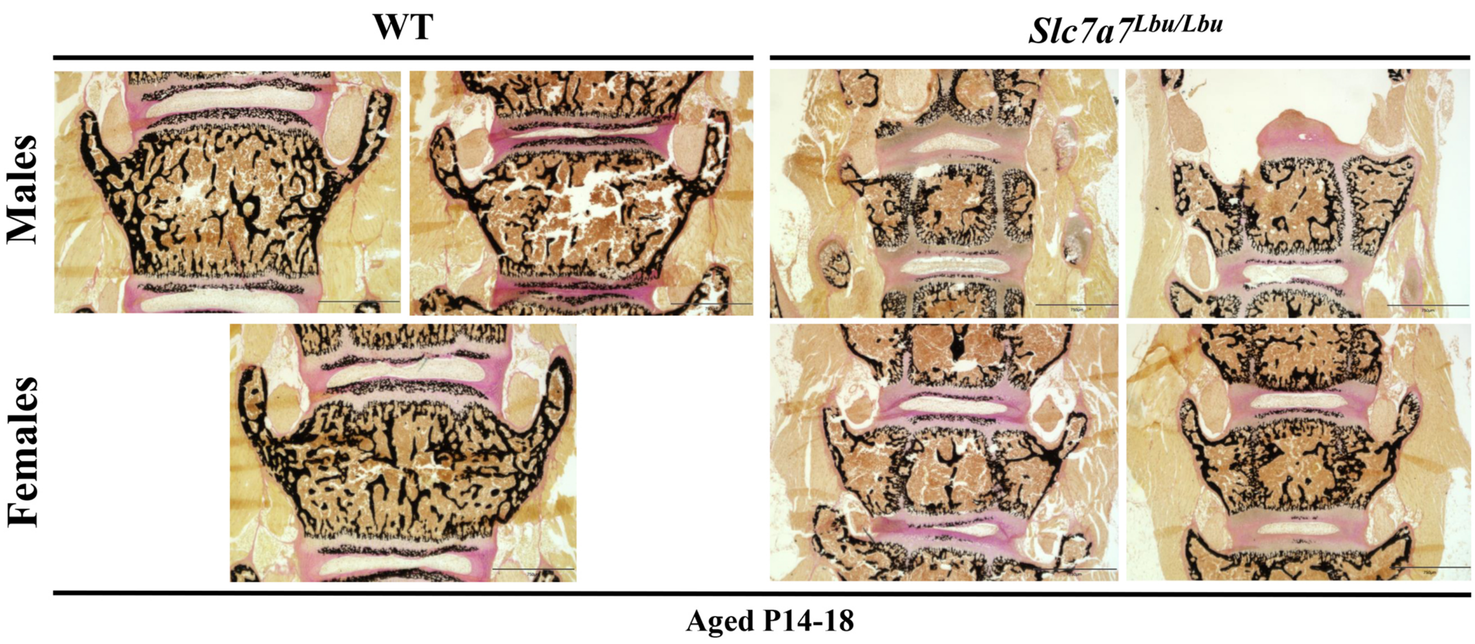

Fig. S3. Variability in the histological architecture of von Kossa-stained L_4 vertebrae sections in WT and *Slc7a7*$^{Lbu/Lbu}$ mice at P14-18. Scale bars indicate 750 µm. WT, wild type.

Disease Models & Mechanisms • Supplementary information

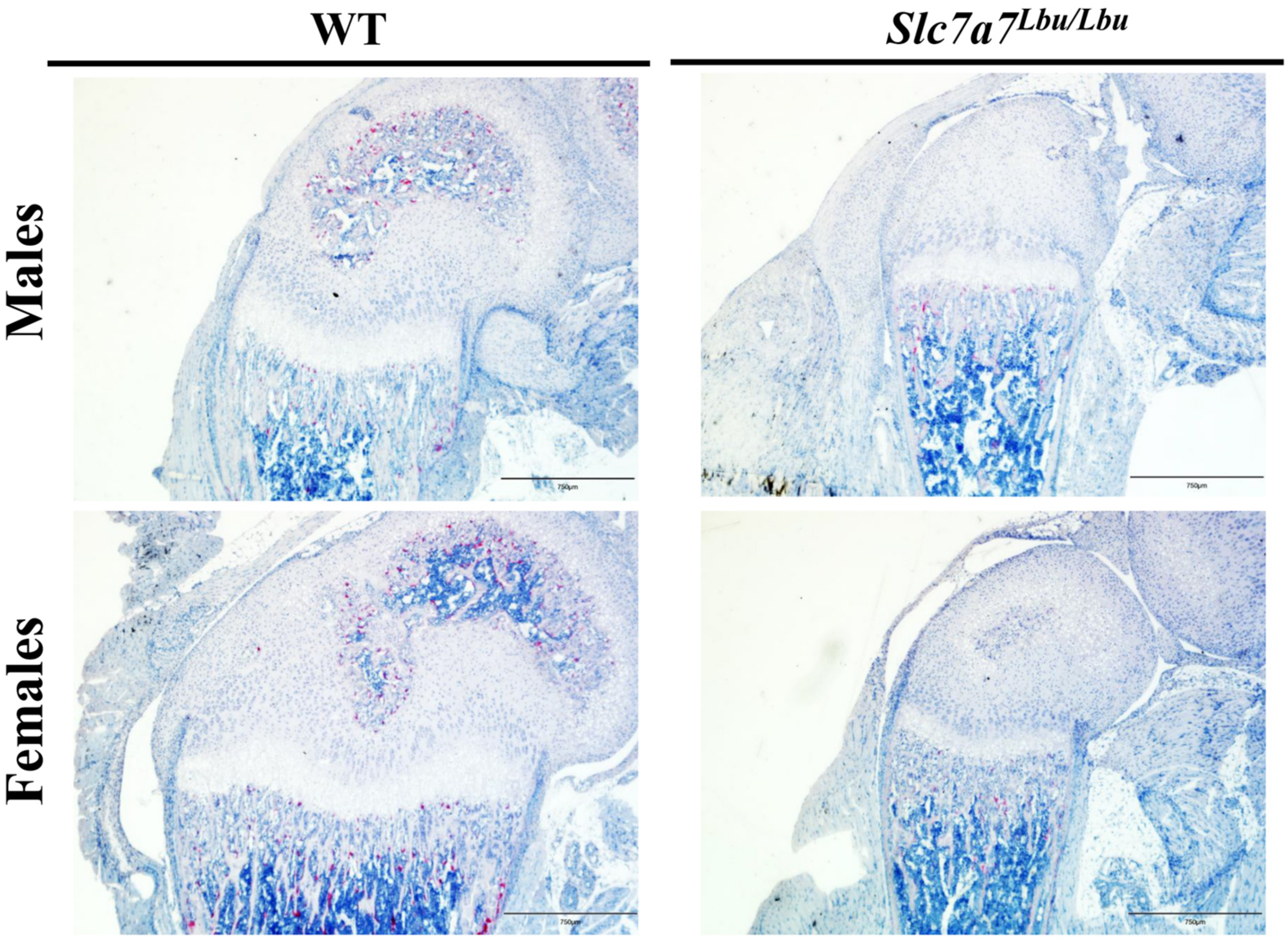

Fig. S4. Variability in the developmental stage of the bone hinders interpretation of histological architecture of tartrate-resistant acid phosphatase (TRAP) staining of lower extremity sections in WT and *Slc7a7*$^{Lbu/Lbu}$ mice at P14-18. Scale bars indicate 750 µm. WT, wild type.

Disease Models & Mechanisms • Supplementary information

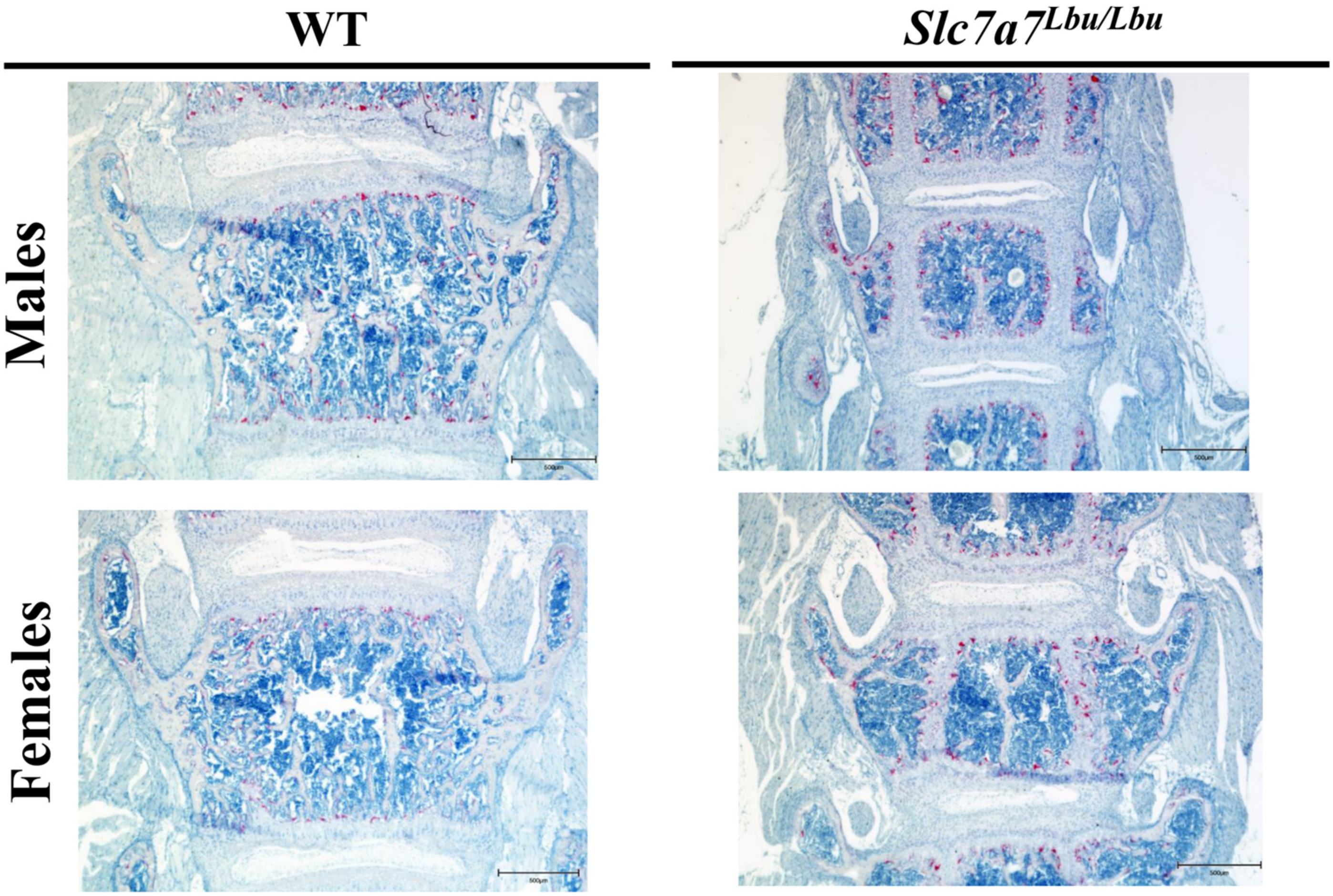

Fig. S5. Variability in the developmental stage of the bone hinders interpretation of histological architecture of tartrate-resistant acid phosphatase (TRAP) staining of vertebral sections in WT and *Slc7a7$^{Lbu/Lbu}$* mice at P14-18. Scale bars indicate 750 µm. WT, wild type.

Disease Models & Mechanisms • Supplementary information

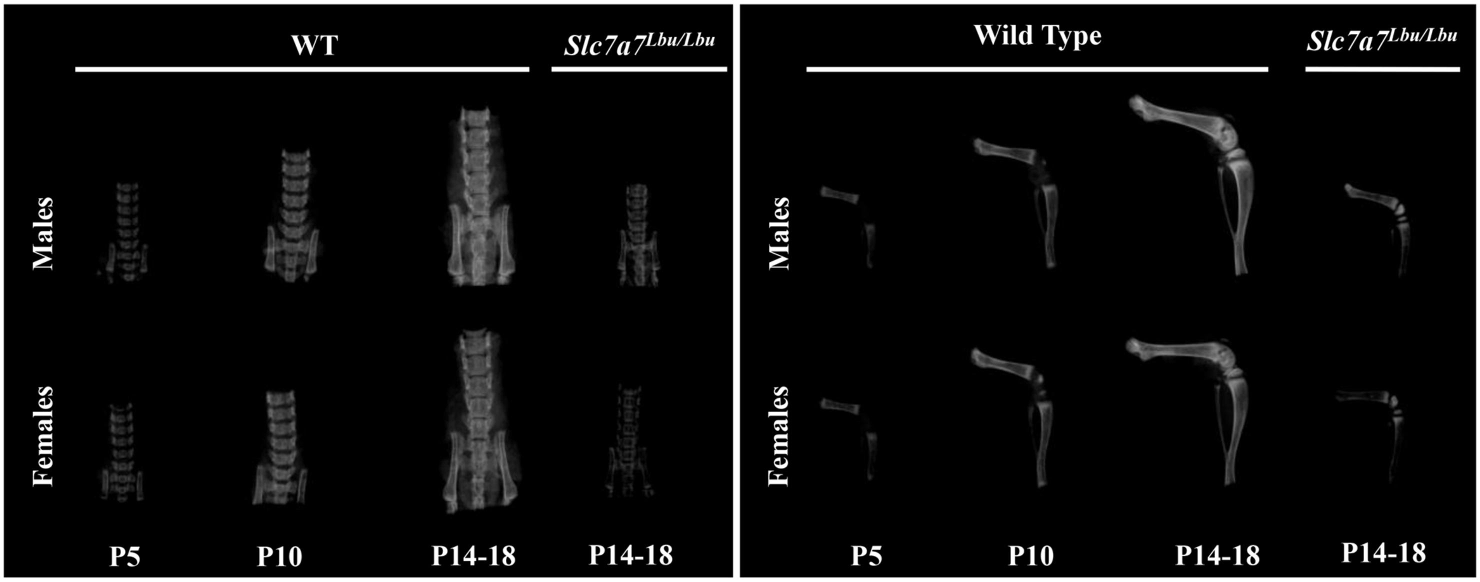

Fig. S6. Representative skeletal radiographs of the spines and lower extremities of male and female WT mice (aged P5, P10, or P14-18) and *Slc7a7*$^{Lbu/Lbu}$ mice (aged P14-18). Although the *Slc7a7*$^{Lbu/Lbu}$ mice might exhibit a mineralization defect, conclusions are hindered by the findings of delayed skeletal development. WT, wild type.

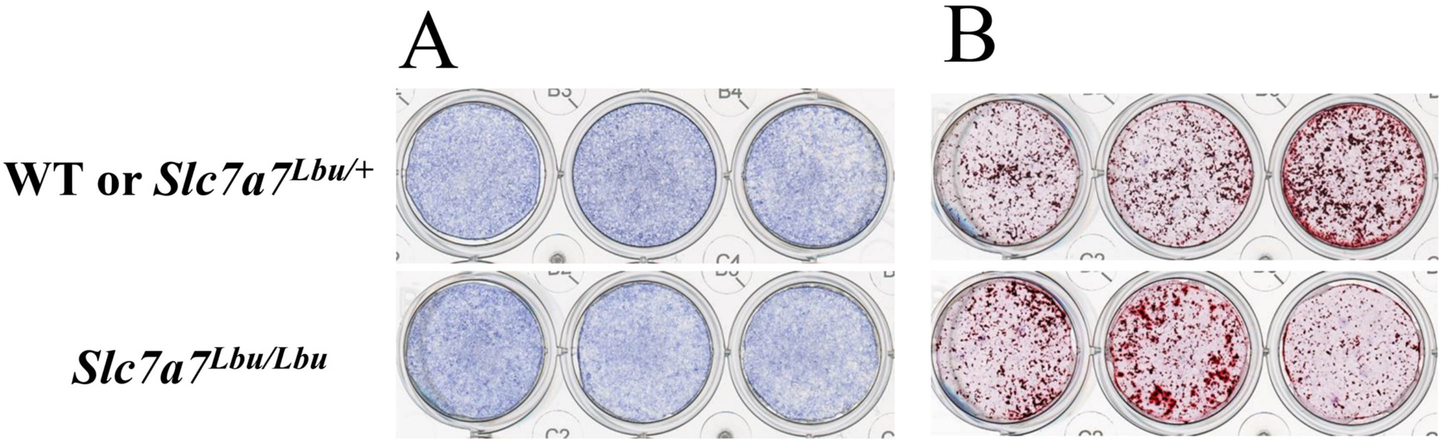

Fig. S7. Osteoblast differentiation and mineralization are similar in calvaria osteoblasts from WT or heterozygous embryos vs. *Slc7a7*$^{Lbu/Lbu}$ (homozygous). (A) Alkaline phosphatase staining was performed after culturing the calvaria osteoblasts in differentiation media for 7 days. (B) Alizarin red staining was performed after culturing calvaria osteoblasts in differentiation media for 15 days. Each well represents cells derived from an individual embryo. WT, wild type.

Disease Models & Mechanisms • Supplementary information

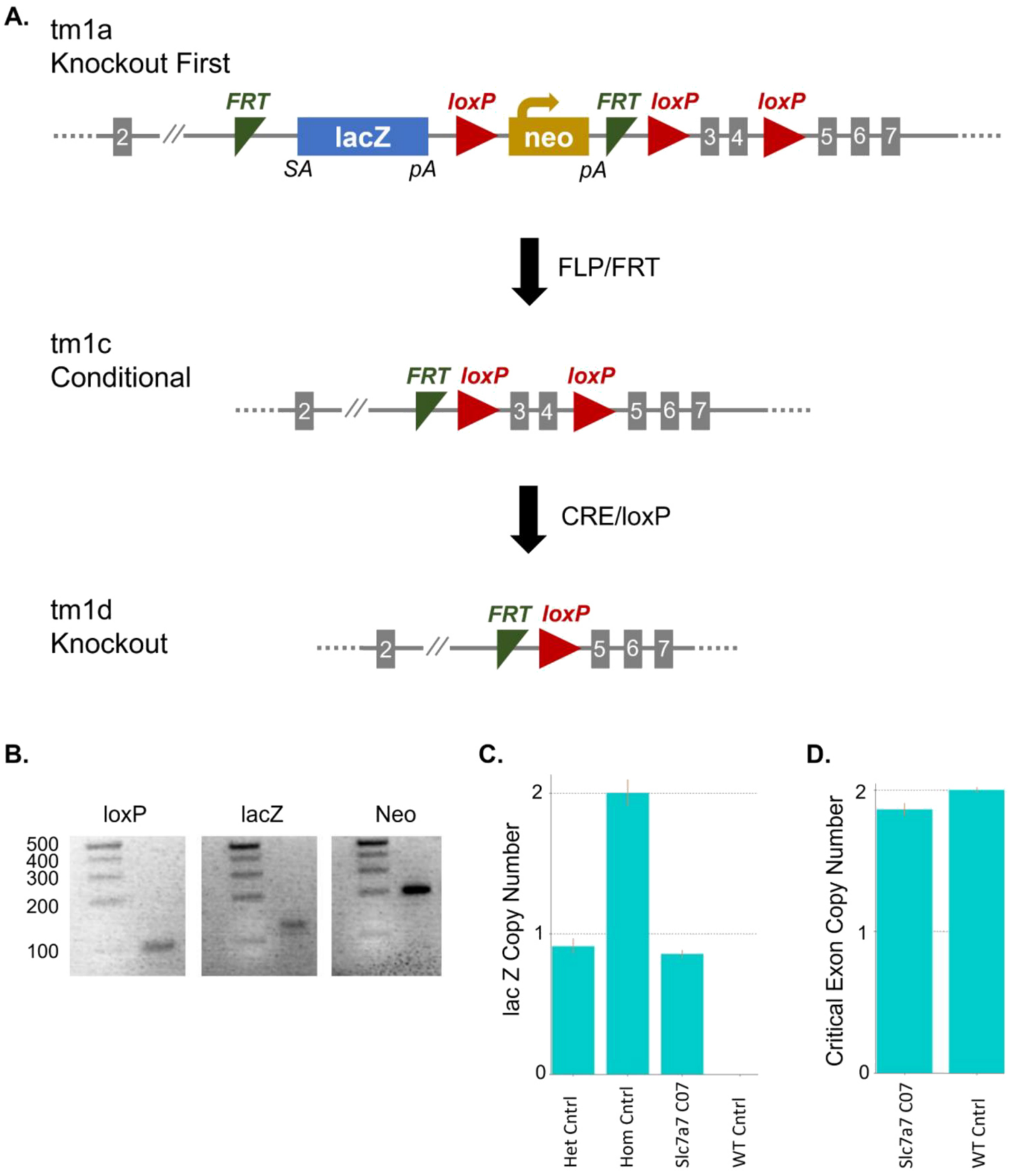
A.
tm1a
Knockout First
FRT
loxP
FRT
loxP
loxP
lacZ
neo
SA
pA
pA
FLP/FRT
tm1c
Conditional
FRT
loxP
loxP
CRE/loxP
tm1d
Knockout
FRT
loxP
B.
loxP
lacZ
Neo
500
400
300
200
100
C.
lac Z Copy Number
Het Cntrl
Hom Cntrl
Slc7a7 C07
WT Cntrl
D.
Critical Exon Copy Number
Slc7a7 C07
WT Cntrl

Fig. S8. Generation of conditional *Slc7a7* mouse model. (A) Diagram of the *Slc7a7* tm1a (knockout first), tm1c (conditional), and tm1d (knockout) alleles. Exons 3 and 4 (ENSMUSE00000124135 and ENSMUSE00000124133 of Ensembl transcript ENSMUST00000000984) are flanked by loxP sites. FLP-mediated recombination of the FRT sites removes the lacZ/Neo insertion to generate the tm1c allele. Cre-mediated loxP recombination removes exons 3 and 4 to generate the tm1d allele. Splicing of exon 2 into 5 is predicted to introduce a frameshift, premature stop codon, and nonsense-mediated mRNA decay. (B) PCR validation/detection of the 3'loxP site, lacZ insert, and Neo cassette insert in ES cell clone EPD0803_3_C07. Values on the y-axis refer to the number of base pairs. (C) Quantitative PCR-based copy number counting for the lacZ insert in ES cell clone EPD0803_3_C07. An established tm1a mouse line (C57BL/6N-*Prdm14*$^{tm1a(EUCOMM)Wtsi}$) was used for the WT, heterozygous, and homozygous tm1a controls. (D) Quantitative PCR-based copy number counting for the critical genomic region (loxP-flanked region) of the tm1a allele in clone EPD0803_3_C07. ES cell, embryonic stem cell; FLP, flippase; FRT, flippase recognition target; Het Cntrl, heterozygous control; Hom Cntrl, homozygous control; PCR, polymerase chain reaction; WT, wild type; WT Cntrl, wild type control.

Disease Models & Mechanisms • Supplementary information

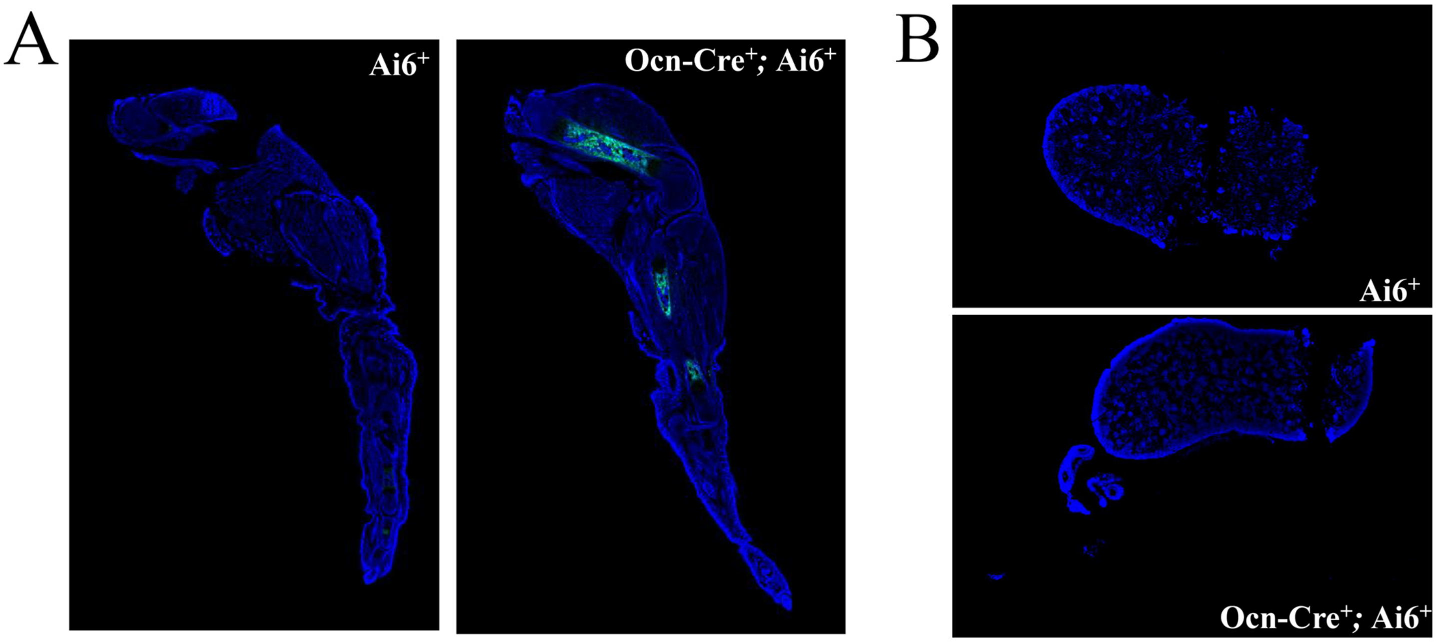

Fig. S9. Positive Ai6 Cre reporter expressing cells within the femurs and tibias harboring the Ocn-Cre transgene. To confirm the activity of our Ocn-Cre, we crossed female $Ai6^+$ Cre reporter mice with male $Ocn\text{-}Cre^+$ mice to generate pups harboring the Ai6 Cre reporter transgene (negative control) and pups harboring both the Ai6 Cre reporter and Ocn-Cre transgenes (positive control). Representative photographs were taken of the lower extremity (A) and renal (B) sections obtained from pups harboring the Ai6 Cre reporter (negative control) or both the Ai6 Cre reporter and Ocn-Cre transgenes (positive control). (A) We observed positive Ai6 Cre reporter expressing cells within the bone tissue and bone marrow of the femur and tibia of the positive control ($Ai6^+$; $Ocn\text{-}Cre^+$) and absent expression of the Ai6 Cre reporter in the lower extremity of the negative control ($Ai6^+$). (B) As expected, we observe no expression of Ai6 Cre reporter in the renal sections of the pups with the $Ai6^+$; $Ocn\text{-}Cre^+$ and $Ai6^+$ genotypes.

Disease Models & Mechanisms • Supplementary information

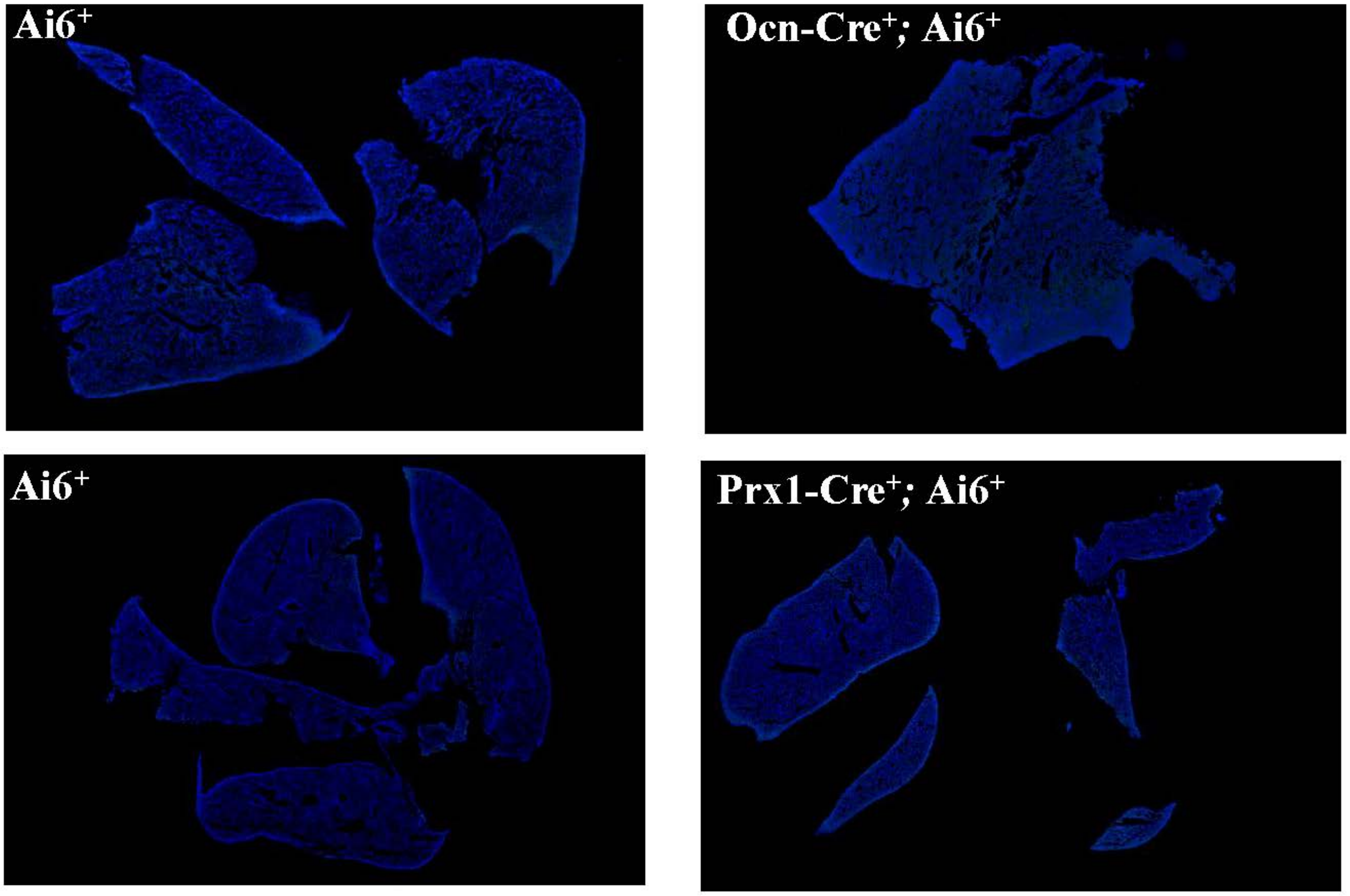

Fig. S10. Negative Ai6 Cre reporter expressing cells within the liver of mice harboring the Ocn-Cre and Prx1-Cre transgene.

Disease Models & Mechanisms • Supplementary information

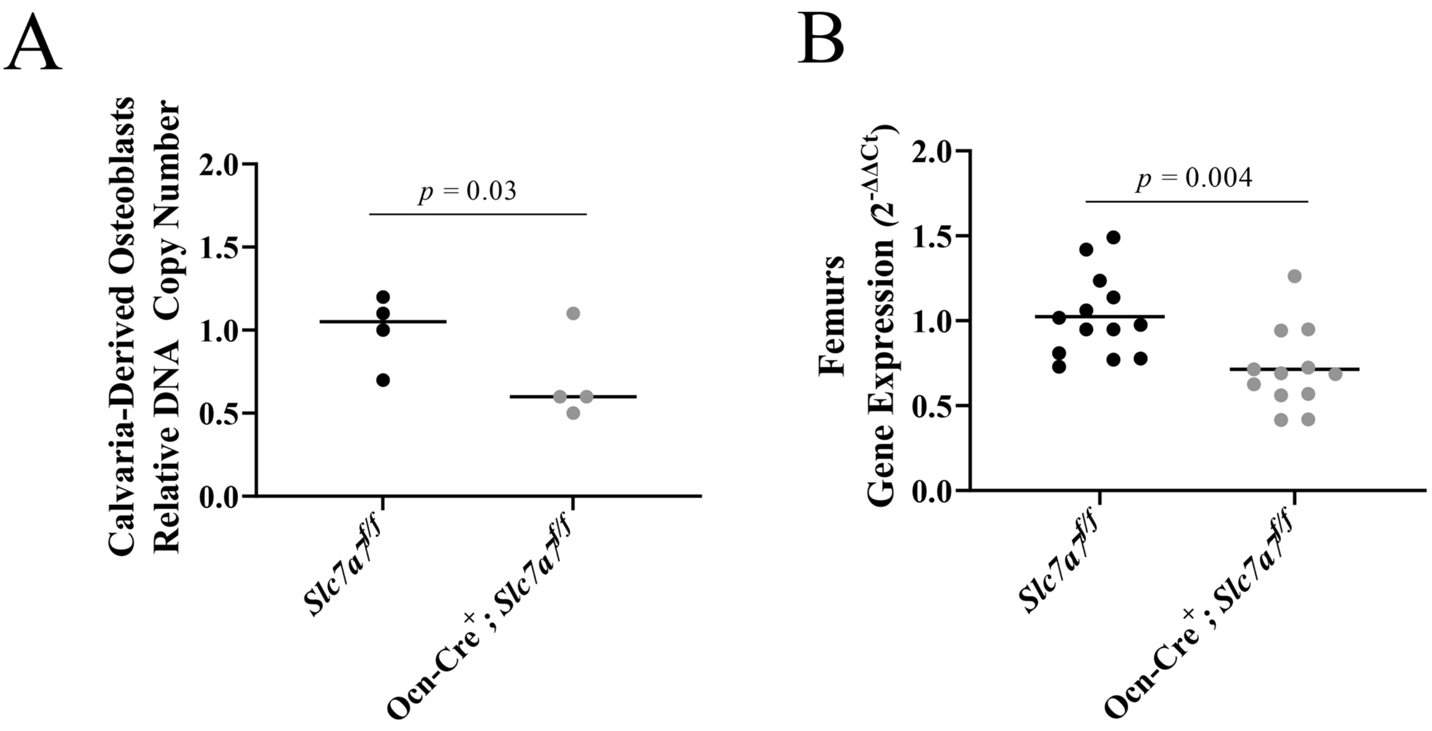

Fig. S11. Assessment of *Slc7a7* deletion in the Ocn-Cre^{+}*; Slc7a7*$^{f/f}$ mouse model. (A) Significant reduction in relative DNA copy number of exon 3 of *Slc7a7* in primary calvaria-derived osteoblast cultures obtained from Ocn-Cre^{+}*;Slc7a7*$^{f/f}$ vs. *Slc7a7*$^{f/f}$ pups (n=4 per genotype). (B) Significant reduction in *Slc7a7* expression in the femurs of adult Ocn-Cre^{+}*; Slc7a7*$^{f/f}$ versus *Slc7a7*$^{f/f}$ mice (n=12 – 13 per genotype). Primers placed in exon 8 (forward) and exon 9 (reverse). Statistical analyses included unpaired two-tailed t-tests.

Disease Models & Mechanisms • Supplementary information

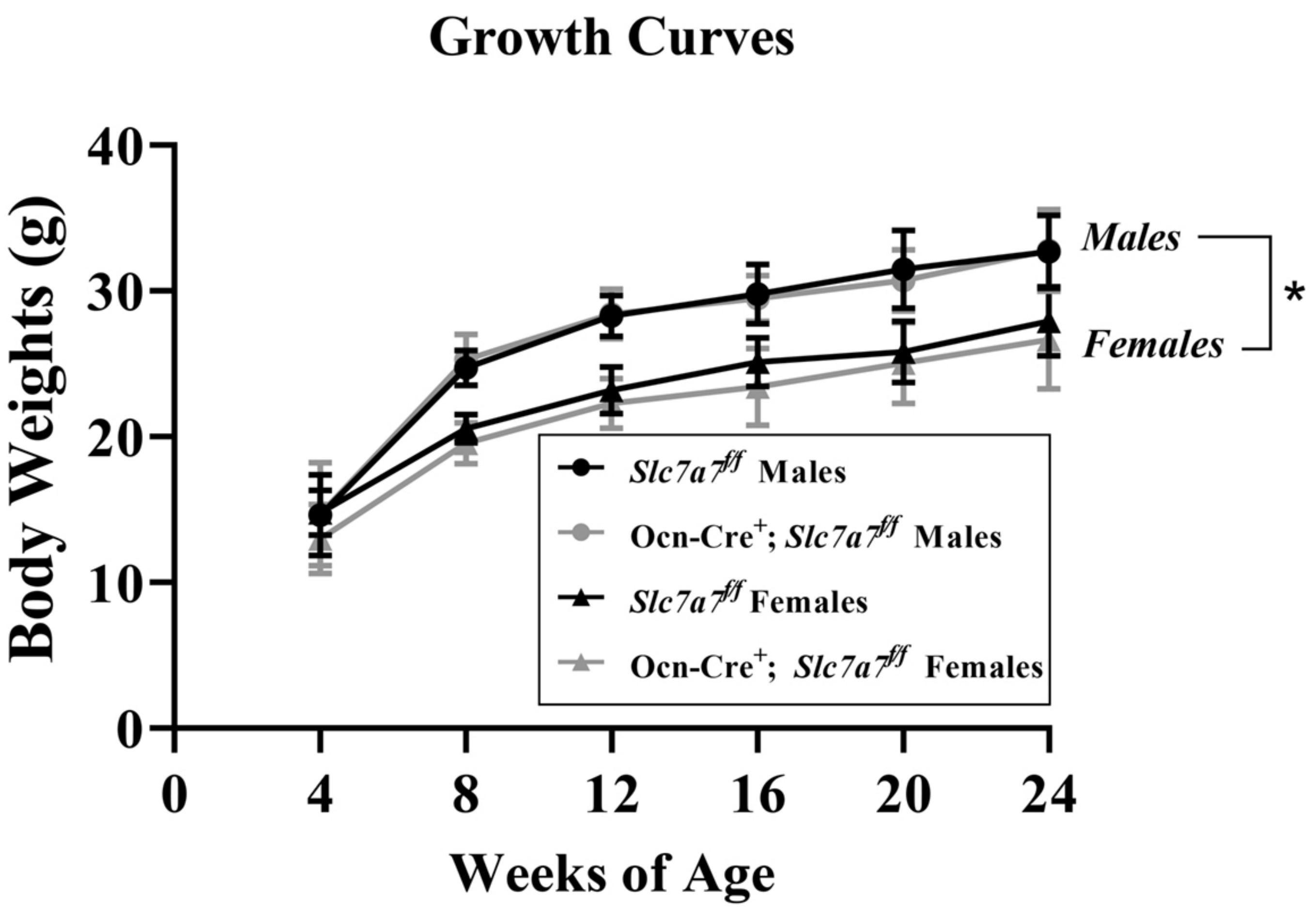

Fig. S12. Similar growth curves in *Slc7a7*$^{f/f}$ and Ocn-Cre^{+}*; Slc7a7*$^{f/f}$ mice (n=8-13 per genotype and sex). Body weights were evaluated in male and female *Slc7a7*$^{f/f}$ and Ocn-Cre^{+}*; Slc7a7*$^{f/f}$ mice from weaning through 24 weeks of age. Statistical analysis included a two-way repeated measures ANOVA with main effects for genotype (*Slc7a7*$^{f/f}$ or Ocn-Cre^{+}*; Slc7a7*$^{f/f}$), sex (male or female), and the genotype by treatment interaction. * indicates $p<0.0001$.

Disease Models & Mechanisms • Supplementary information

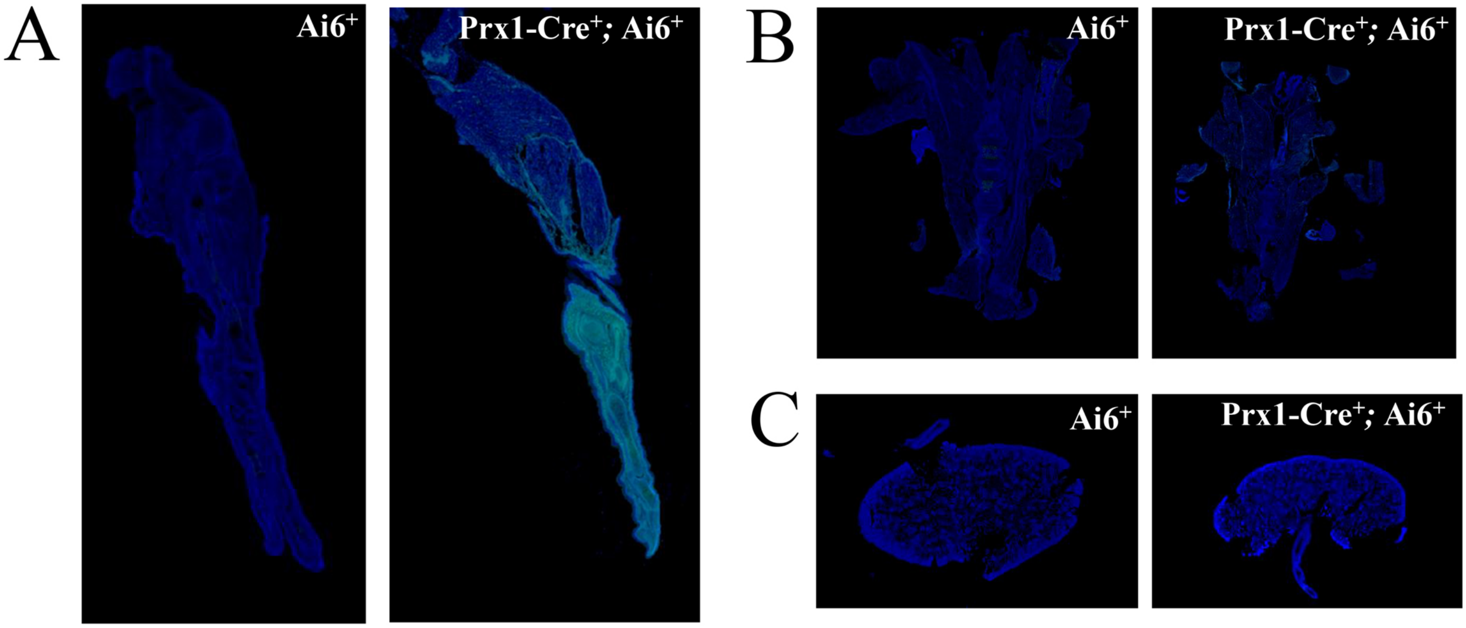

Fig. S13. Positive Ai6 Cre reporter expressing cells within the femurs and tibias harboring the Prx1-Cre transgene. To confirm the activity of our Prx1-Cre, we crossed female Ai6$^+$Cre reporter mice with male Prx1-Cre$^+$ mice to generate pups harboring the Ai6 Cre reporter transgene (negative control) and pups harboring both the Ai6 Cre reporter and Prx1-Cre transgenes (positive control). Representative photographs were taken of the lower extremity (A), spine (B), and renal (C) sections obtained from pups harboring the Ai6 Cre reporter (negative control) or both the Ai6 Cre reporter and Prx1-Cre transgenes (positive control). (A) We observed positive Ai6 Cre reporter expressing cells within the bone tissue and bone marrow of the long bones but not muscle in the positive control (Ai6$^+$; Prx1-Cre$^+$) and absent expression of the Ai6 Cre reporter in the lower extremity of the negative control (Ai6$^+$). (B) As expected, we observe no expression of Ai6 Cre reporter in the spine sections of the pups with the Ai6$^+$; Prx1-Cre$^+$ and Ai6$^+$ genotypes as Prx1 is not expressed in axial skeleton. (C) As expected, we observe no expression of Ai6 Cre reporter in the renal sections of the pups with the Ai6$^+$; Prx1-Cre$^+$ and Ai6$^+$ genotypes.

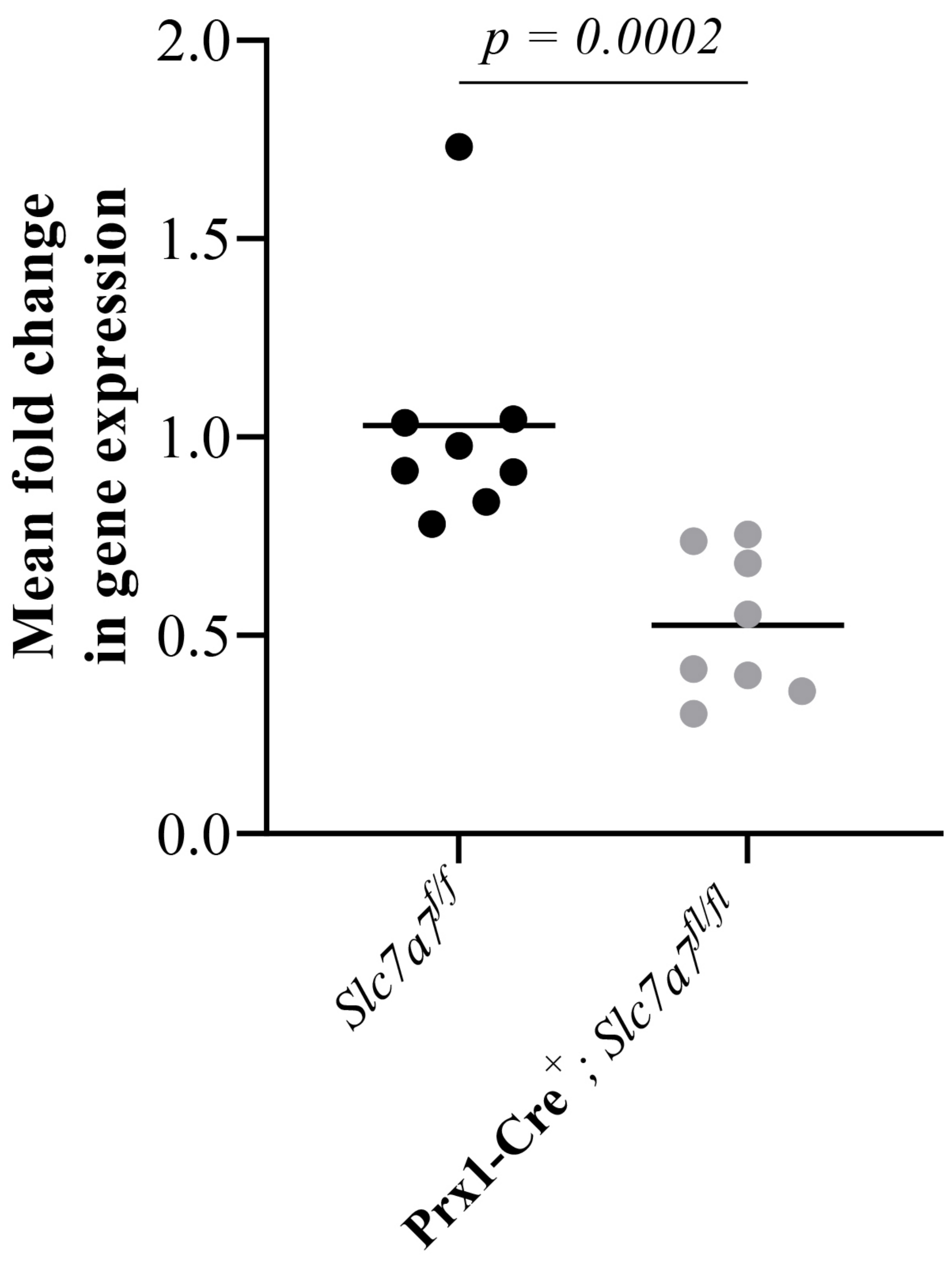

Fig. S14. Significant reduction in intact *Slc7a7* expression in the femurs of adult Prx1-Cre^{+}; *Slc7a7*$^{f/f}$ versus *Slc7a7*$^{f/f}$ mice. Primers were placed in exon 4 (forward) and exon 5 (reverse). Statistical analyses utilized a Mann-Whitney test given the distribution of the data (n=8 per genotype).

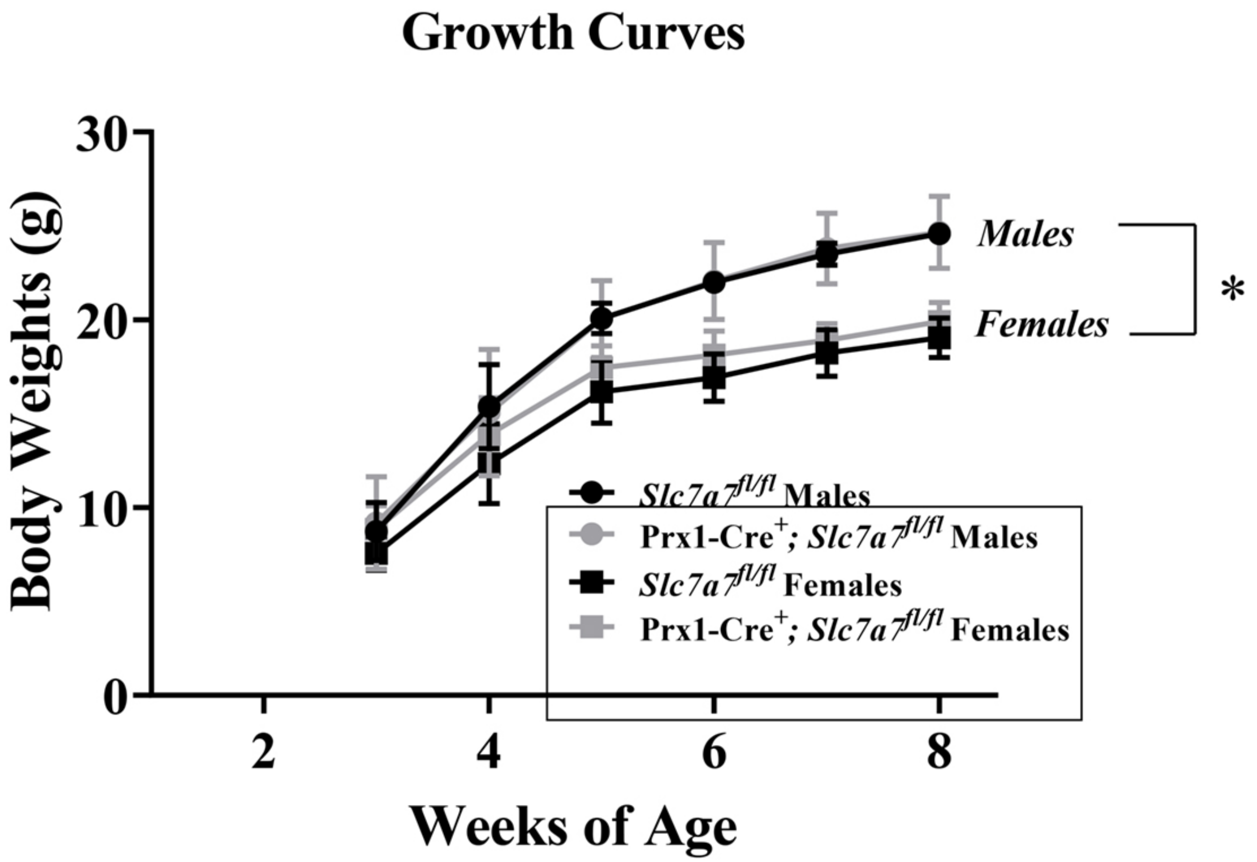

Fig. S15. Similar growth curves in *Slc7a7*$^{f/f}$ and Prx1-Cre^{+}*; Slc7a7*$^{f/f}$ mice. Body weights were evaluated in male and female *Slc7a7*$^{f/f}$ and Prx1-Cre^{+}*; Slc7a7*$^{f/f}$ mice from weaning through 8 weeks of age (n=7-10 per genotype and sex). The means + SD are plotted. Statistical analysis included a two-way repeated measures ANOVA with main effects for genotype (*Slc7a7*$^{f/f}$ or Prx1-Cre^{+}*; Slc7a7*$^{f/f}$), sex (male or female), and the genotype by treatment interaction. * indicates p<0.0001.

Table S1. Standard PCR Primers for Genotyping

**Assay**	**Forward Primer (5'→3')**	**Reverse Primer (5'→3')**	**Product (bp)**
tm1a 3'loxP	GAGATGGCGCAACGCAATTAA	GGCGAGCTCAGACCATAACTT	71
tm1a lacZ	ATCACGACGCGCTGTATC	ACATCGGGCAAATAATATCG	108
tm1a Neo	GGCCGCTTTTCTGGATTCAT	AGTCCCGCTCAGAAGAACTC	203
tm1a to tm1c conversion	AAGGCGCATAACGATACCAC	CCGCCTACTGCGACTATAGAGA	tm1a: 7122<br>tm1c: 218
tm1(FLP1)Dym/J	CACTGATATTGTAAGTAGTTTGC	CTAGTGCGAAGTAGTGATCAGG	725
tm1c	GGGCTGGAAGTTTGGTCTCT	ACACTGACCGTGACCTACCC	WT: 483<br>tm1c: 665
Ocn-Cre	CAAATAGCCCTGGCAGAT	TGATACAAGGGACATCTTCC	WT: 386<br>Ocn-Cre: 300
Prx1-Cre	GTCATGAAAACACCGTCCAG	CATCGACCGGTAATGCAG	Prx1-Cre: 1121
Universal Cre	TCCAATTTACTGACCGTACACCAA	CCTGATCCTGGCAATTTCGGCTA	500

WT, wild type

Disease Models & Mechanisms • Supplementary information

Table S2. Quantitative PCR Primers for Gene Expression Studies. The *Slc7a7* primers in exon 8 and 9 were used for Ocn-Cre studies and the primers in exon 4 and 5 were used for Prx-1 Cre studies.

Gene	Direction	Sequence
B2m	Forward	GGTCTTTCTGGTGCTTGTC
B2m	Reverse	CGTATGTATCAGTCTCAGT
Bglap	Forward	GGCTTTTGATAATTGACTGGGCTGAACACT
Bglap	Reverse	CACCTGAGGGCTGCTGCAAAGATTGATTAG
Col1a1	Forward	TTGGGGCAAGACAGTCATCGAAT
Col1a1	Reverse	TTGGGGTGGAGGGAGTTTACACGAA
Ghr	Forward	TCTGGAAAGCCTCGATTCAC
Ghr	Reverse	TCAGGGCATTCTTTCCATTC
Igf1	Forward	GCTGGTGGATGCTCTTCAGT
Igf1	Reverse	TCCGGAAGCAACACTCATCC
Igfbp4	Forward	TACCCACGAAGACCTCTTCATC
Igfbp4	Reverse	GTCTTCCGATCCACACACCA
Igf1r	Forward	ATGGCTTCGTTATCCACGAC
Igf1r	Reverse	AATGGCGGATCTTCACGTAG
Opg	Forward	CCAACAGTTTATCCAGCTGTCATGTC
Opg	Reverse	CCATATTCAAAGAGATCCAATGGAGTCTA
Osx	Forward	ATCGGGGCGGCTGATTG
Osx	Reverse	GGGCGTTCTACCTGCGACTG
Rpl7	Forward	CCCTGAAGACACTTCGAAAGG
Rpl7	Reverse	GCTTTCCTTGCCATCCTGGC
Slc7a7 (exon 8)	Forward	TGTGGGCCTTTCTATTGTG
Slc7a7 (exon 9)	Reverse	AGGGAGTTGATGGTGTCGCTGTAA
Slc7a7 (exon 4)	Forward	CAGCCCTGTTCTCCTACTCG
Slc7a7 (exon 5)	Reverse	GGAAATGCCAATGGAGAGG
Tnfsf11	Forward	GGCTATGTCAGCTCCTAAAGTCAA
Tnfsf11	Reverse	CCCTGAAGGTACTCGTAGCTAAG
Trap	Forward	CTGGTATGTGCTGGCTGGAA
Trap	Reverse	GTAAGGGCTGGGGAAGTTCC
